# Staudinger Reaction-Responsive Coacervates for Cytosolic Antibody Delivery and TRIM21-Mediated Protein Degradation

**DOI:** 10.1021/jacs.4c17054

**Published:** 2025-01-13

**Authors:** Yishu Bao, Zhiyi Xu, Kai Cheng, Xiaojing Li, Fangke Chen, Dingdong Yuan, Fang Zhang, Audrey Run-Yu Che, Xiangze Zeng, Yuan-Di Zhao, Jiang Xia

**Affiliations:** † Department of Chemistry, The Chinese University of Hong Kong, Shatin, Hong Kong SAR 99999, China; ‡ Department of Physics, Hong Kong Baptist University, Kowloon Tong, Hong Kong SAR 99999, China; § Britton Chance Center for Biomedical Photonics at Wuhan National Laboratory for Optoelectronics-Hubei Bioinformatics and Molecular Imaging Key Laboratory, Department of Biomedical Engineering, College of Life Science and Technology, Huazhong University of Science and Technology, Wuhan 430074, Hubei, China; ∥ Department of Natural Sciences, Pitzer and Scripps Colleges, 925 N. Mills Ave, Claremont, California 91711, United States

## Abstract

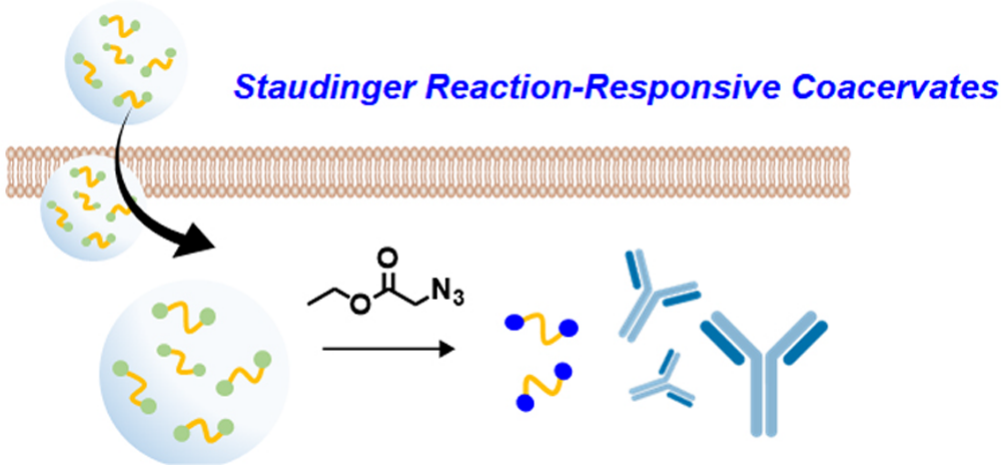

A low-molecular-weight compound whose structure strikes a fine balance between hydrophobicity and hydrophilicity may form coacervates via liquid–liquid phase separation in an aqueous solution. These coacervates may encapsulate and convoy proteins across the plasma membrane into the cell. However, releasing the cargo from the vehicle to the cytosol is challenging. Here, we address this issue by designing phase-separating coacervates, which are disassembled by the bioorthogonal Staudinger reaction. We constructed and selected triphenylphosphine-based compounds that formed phase-separated coacervates in an aqueous solution. Reacting the coacervates with azides resulted in microdroplet dissolution, so they received the name Staudinger Reaction-Responsive Coacervates, **SR-Coa**. **SR-Coa** could encapsulate proteins, including antibodies, and translocate them across the plasma membrane into the cell. Further treatment of the cell with ethyl azidoacetate induced the cargo dispersion from the puncta to the cytosolic distribution. We showcased an application of the **SR-Coa**/ethyl azidoacetate system in facilitating the translocation of the EGFR/antibody complex into the cell, which induced EGFR degradation via the TRIM21-dependent pathway both in vitro and in vivo. Besides the membrane protein EGFR, this system could also degrade endogenous protein EZH2. Taken together, here we report a strategy of controlling molecular coacervates by a bioorthogonal reaction in the cell for cytosolic protein delivery and demonstrate its use in promoting targeted protein degradation via the proteasome-dependent pathway.

## Significance Statement

Molecular coacervates are known protein carriers: they can encapsulate proteins and deliver them into cells. However, the protein cargo must be released into the cytosol to perform its function. Here, we designed triphenylphosphine-based compounds to form molecular coacervates through liquid–liquid phase separation, showed intracellular protein delivery and release by an azide, and demonstrated an application in targeted protein degradation via the TRIM21-mediated pathway.

## Introduction

Proteins are critical regulators of a wide range of cellular processes, including signal transduction, gene expression, cell movement, and metabolism. Molecules that block, intervene, or amend the function of proteins inside cells are of tremendous value in chemical biology and therapies. Small molecule compounds are by far the most effective class of therapeutics for intracellular protein targets. Initially used for enzyme inhibition, they are increasingly being employed to target nonenzymatic proteins. For example, proteolysis-targeting chimera (PROTAC) degraders are compounds that harness the ubiquitin–proteasome system to degrade selected proteins; their success has moved targeted protein degradation (TPD) from academia to industry.^1−4^ Notwithstanding, discovering compounds that bind selectively to the target protein is not trivial. Alternatively, gene therapies using plasmids or viral vectors have caught attention, but concerns regarding the safety of gene integration into the genome are unsettled.^5,6^ In light of these limitations, protein therapeutics empowered by cytosolic translation tools are emerging as an alternative to compound- or gene-based approaches.

Targeted protein degradation is a viable method of interfering with cell function. Besides using PROTAC to degrade protein targets inside cells, outside cells, or on the membrane, researchers also use antibodies to precisely recognize protein targets and bring them to the cellular disposal machinery: the proteasome or the lysosome.^7−17^ Among these strategies, Trim-Away is a unique one as it requires only a natural protein TRIM21, which is an E3 ubiquitin ligase that binds with high affinity to the Fc domain of antibodies, which are widely expressed in diverse cell types and tissues. After the introduction of exogenous TRIM21 and an antibody against the protein of interest, TRIM21-mediated ubiquitination led to the degradation of the antibody-bound protein of interest within minutes of application.^18^ Therefore, the Trim-Away approach can acutely and rapidly degrade endogenous proteins without the prior modification of either the antibody or the TRIM21 protein. However, translocating the antibody or the antibody–antigen complex into the cell where TRIM21 and proteasomes reside is challenging. In this report, the authors used electroporation to facilitate the translocation of the antibody or antibody–antigen complex into the cell.^18^ Such a physical method limits the use of Trim-Away degraders. Here, we seek to develop a method for translocating the antibody or antibody–antigen complex into the cytosol based on small-molecule coacervates.

Delivering protein cargo across the plasma membrane while maintaining their activity is a prerequisite for proteins to take effect inside cells.^19^ Despite tremendous advancements in recent years, intracellular delivery systems are still imperfect.^20^ Nanosized carriers, such as synthetic nanoparticles^21−25^ and nature-borne extracellular vesicles,^26,27^ have been used in cytosolic protein delivery. Cell-penetrating peptides or membrane-permeabilizing peptides represent the most recent progress of peptide-based tools for delivering proteins into cells.^27−34^ Notwithstanding, in most of these systems, peptides with long or complex sequences or a high concentration of peptides are required to facilitate protein delivery, which is often inconvenient in practical uses.

On another note, researchers discovered that molecules in the form of phase-separated coacervates may spontaneously enter cells. Molecules with certain structural features can undergo liquid–liquid phase separation (LLPS) and form microdroplets called coacervates. The coacervate state endows the molecule with new properties that are unattainable in other states including discrete solutions, amorphous precipitates, or crystalline states. For example, the interaction between coacervates and biological membranes can lead to their spontaneous entry into the liposome.^35,36^ This observation raised the speculation that some coacervates may enter the cells. This was first proven using polymers: cationic polymers were shown to deliver the anionic heparin, and amylose-based coacervates could deliver myoglobin into human mesenchymal stem cells.^37,38^ A sophisticated peptide dendrimer can coassemble with proteins for cytosolic translocation. Futaki and co-workers showed that an Fc region binding peptide conjugated with attenuated cationic amphiphilic lytic peptide L17E trimer [FcB-(L17E)_3_] could deliver immunoglobulin G (IgG) into cells by forming particle-like liquid droplets.^39^ These examples proved the feasibility of coacervate-mediated delivery, but the cargo protein may still remain encapsulated within the microdroplets in the cell, disallowing them to interact with their targets in the cytosol. Miserez and co-workers designed a phase-separating peptide HBpep-SR that can release protein cargo by reacting with intracellular glutathione: in the cytosol, glutathione-mediated reduction removes the side chain group on lysine, disassembles the phase-separated coacervates, and releases the payload.^40−42^

In addition to proteins and peptides, we have developed low-molecular-weight small molecules that can form coacervates, convoy proteins into cells, and use chemical reactions to regulate cargo distribution inside the cell. We designed a photoresponsive, phase-separating fluorescent molecule (PPFM) with a molecular weight of 666.6 Da based on pyrene that can (1) undergo LLPS in the aqueous solution, (2) carry payloads into cells, (3) respond to a benign stimulus, a 405 nm light, and (4) show a fast response within minutes.^43^ Notwithstanding, the PPFM compound is not flawless. The photolytic reaction generates a thioaldehyde compound in the cell, which may cross-link proteins and alter cellular physiology in the long run, although the short-term cytotoxicity was shown to be minimal. Also, the 405 nm UV light is not optimal, but efforts to design molecules that respond to longer light wavelengths were unfruitful.

**Figure 1:**
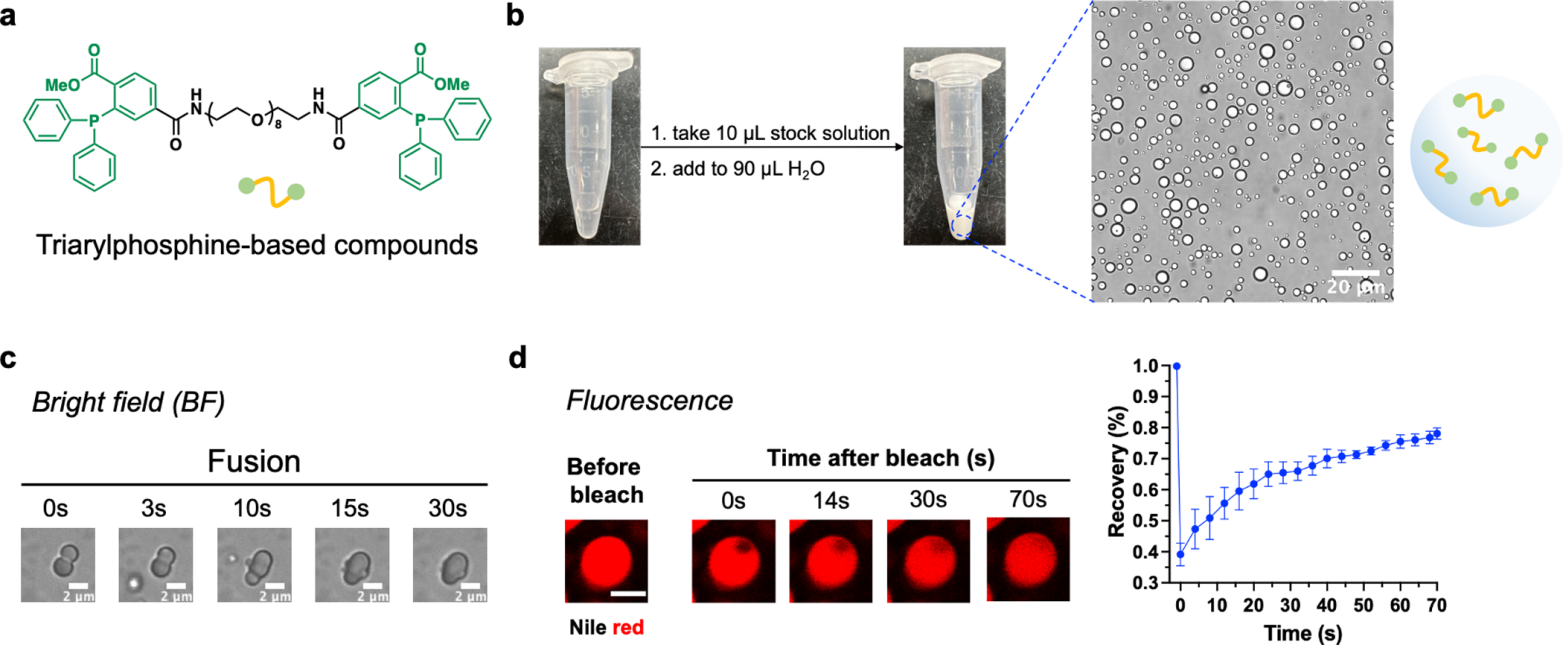
Triphenylphosphine-based phase-separating compounds. (a) Chemical structure of the triarylphosphine compounds. (b) Confocal microscopic images of the microdroplets formed by triphenylphosphine-based compounds (10 mg/mL in a 1:10 DMSO/water solution). (c) Confocal microscopic images showing the fusion of microdroplets. (d) Fluorescence recovery after photobleaching (FRAP) analysis showed the recovery of fluorescence after photobleaching. The fluorescence of Nile red was detected using the excitation/emission wavelengths of 549 nm/628 nm. Data are presented as the mean ± s.d. of *n* = 3 independent experiments.

Here, we aim to dissipate molecular coacervation using a different stimulus, a small molecule compound, through a meticulously designed release mechanism, a biocompatible reaction. We reason that triarylphosphine and the azide-reaction product triarylphosphine oxide have a subtle difference in hydrophobicity, which may allow the azide reaction to disassemble triarylphosphine-based coacervates. Therefore, we designed and synthesized triarylphosphine-based phase-separating molecules, demonstrated their azide responsiveness and intracellular delivery capability, and showcased one application of degrading target proteins via the TRIM21-mediated ubiquitination pathway both in vitro and in vivo (Figure 1).

## Results

### Triarylphosphine-Based Phase-Separating Compounds

Bioorthogonal reactions, such as Staudinger ligation,^44,45^ copper-catalyzed azide–alkyne cycloaddition (CuAAC),^46^ strain-promoted azide–alkyne cycloaddition (SPAAC),^47^ and inverse-electron-demand Diels–Alder (IEDDA) cycloaddition,^48^ have provided powerful tools for manipulating biological processes within living systems.^49−51^ The Staudinger reaction between triarylphosphine and azides forms a triarylphosphine oxide. Azides are well tolerated in biological systems^52−55^; for example, Zidovudine, an antiretroviral drug, contains an azide. Therefore, we envision that we can design molecular coacervates based on triarylphosphine and use azide to disassemble the coacervates.

Dimeric triphenylphosphine-based molecules were designed based on the “sticker-and-spacer” model. Polyethylene glycol (PEG) spacers with various lengths were screened including PEG_2_, PEG_4_, PEG_6_, and PEG_8_ to link two triphenylphosphine groups. These compounds were first dissolved in DMSO as stock solutions; the stock solutions were then diluted in water to observe the compound states in the solution. Triphenylphosphine compounds with PEG_2_, PEG_4_, and PEG_6_ linkers formed aggregates, irregular droplets, and hollow droplets in the aqueous solution, respectively (Figure S1). Only the compound with a PEG_8_ linker gave regular, globular microdroplets with sizes between 1 and 10 μm (Figures 1a,b and S2). The zeta potential of the coacervates switches from positive to negative at pH 11, likely due to the absorption of OH^–^ ions on the droplet’s surface or the ionization of the amide bond.^56,57^ Spontaneous fusion of the microdroplets was observed (Figure 1c). The microdroplets can also be labeled with Nile red. After photobleaching an area within the microdroplet, the fluorescent signal immediately decreased and gradually recovered to about 80% in 70 s (Figure 1d). Both results show that the microdroplets have liquid-like properties, meeting the features of phase-separated coacervates. Cationic and zwitterionic dyes could be encapsulated or coassembled within the coacervates, but an anionic dye, 5-tetramethylrhodamine (5-FAM), showed a low recruitment efficiency. The dye structure also affected the droplet size: for unknown reasons, Cy3-encapsulated coacervates showed much smaller diameters (Figures S3 and S4a). In addition, the coacervates also efficiently recruit proteins (Figure S4b). Taken together, we show for the first time that triphenylphosphine, a component of the Staudinger reaction, can be used as a sticker to prepare phase-separating molecules with an appropriate PEG linker. The triphenylphosphine-based coacervates could also enrich organic dyes and proteins.

### Staudinger Reaction-Responsive Coacervate Disintegration

**Figure 2:**
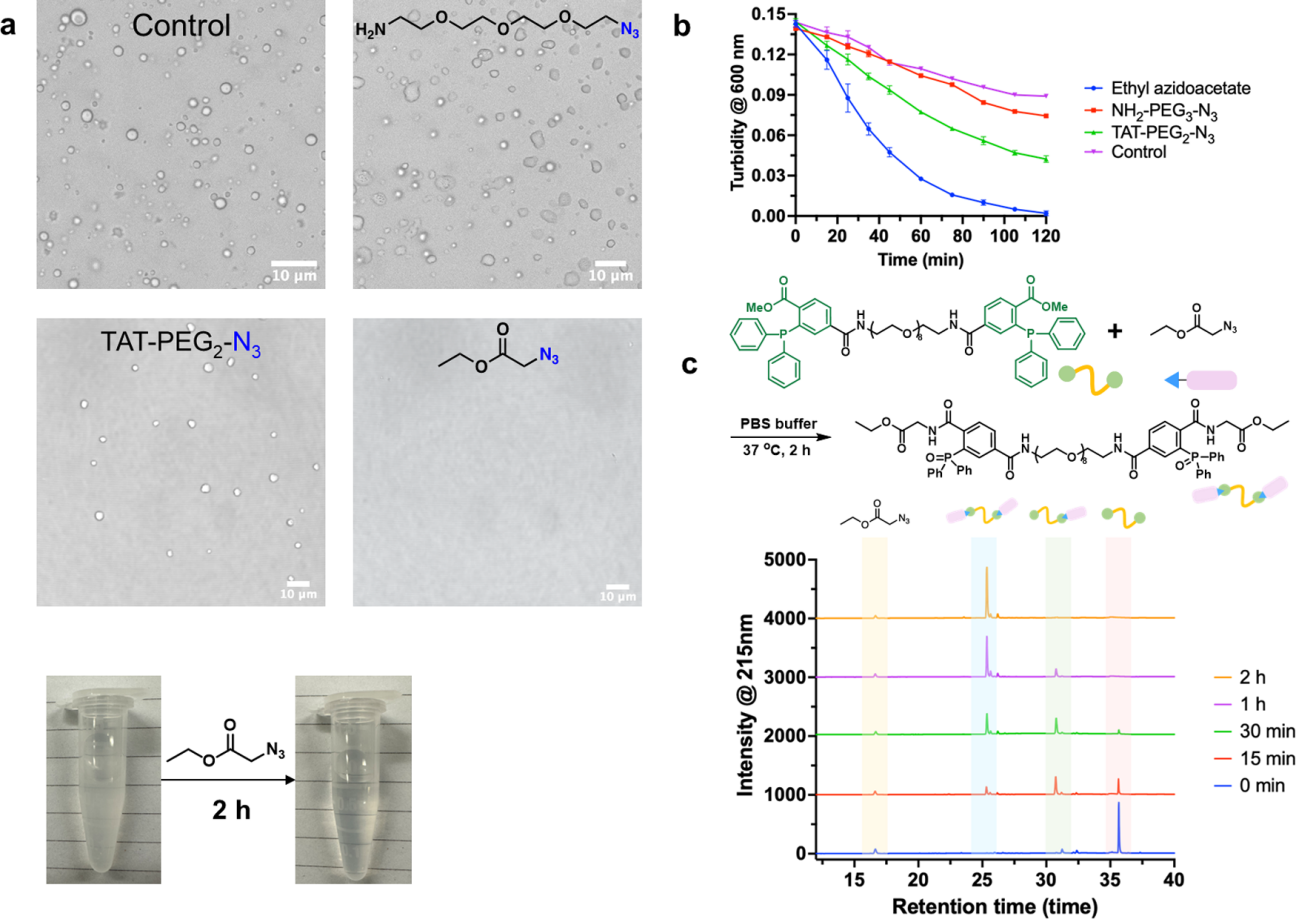
Staudinger reaction-responsive coacervate disintegration. (a) Microscopic images of coacervate solutions after reaction with different azides. (b) Turbidity of reaction solutions of the coacervates and azide-containing compounds (the signal of the blank solvent was deducted). TAT peptides: a cell-penetrating peptide (sequence: GRKKRRQRRRPQ). (c) RP-HPLC chromatograms of the reaction were obtained in PBS buffer. The concentrations of triphenylphosphine compound and ethyl azidoacetate were 90 and 450 μM, respectively. The gradual disappearance of the triphenylphosphine compound was accompanied by the increase of mono- and dioxide products.

Next, we explored whether azides could disintegrate the coacervates via the Staudinger reaction. After screening a set of small molecule azides and a peptide azide (Figure S5), we focused on three azide-containing compounds: an azide-labeled TAT peptide, amine-PEG_3_-azide, and ethyl azidoacetate (Figure 2). Confocal images showed that ethyl azidoacetate could disintegrate the coacervates efficiently, while TAT peptides and amine-PEG_3_-azide could not (Figure 2a). All three compounds caused a decrease in the turbidity of the coacervate solution, with the ethyl azidoacetate treatment showing the most drastic decrease to almost zero in 2 h (Figure 2b). We speculate that the azide-labeled TAT peptide and amine-PEG_3_-azide introduce positive charges into the products. The resultant cation-π interactions may explain why they could not disintegrate the coacervates. In contrast, the triphenylphosphine oxide product of ethyl azidoacetate and the formation of the amide bond increased the hydrophilicity. This hypothesis was supported by an experiment comparing the contact angle of the solution before and after reacting with ethyl azidoacetate. The triarylphosphine oxide product showed a smaller contact angle than the reactant, suggesting that the azide reaction increased the molecule’s hydrophilicity, in line with the observation that the azide reaction dissipates the coacervates (Figure S6). A computational study also supported the difference between triphenylphosphine and triphenylphosphine oxide in hydrophobicity. The hydration-free energy of the amide-bearing triphenylphosphine oxide was calculated to be −18.9 kcal/mol, lower than that of triphenylphosphine (−7.3 kcal/mol) (Figure S7), suggesting a significant decrease in the hydrophobicity of the triphenylphosphine structure after the Staudinger reaction. Hydrogen peroxides could decompose the coacervates, which implies that hydrogen bonds contribute to **SR-Coa** formation (Figure S8). The reaction was also monitored by RP-HPLC (Figures 2c and S9). Incubation of the triphenylphosphine-based compound and ethyl azidoacetate for 30 min converted over 90% of the compound to mono-oxide or dioxide. The oxide formation was completed after 2 h, which was also confirmed by NMR and ESI-MS (Figure S10). Consequently, the turbid solution became clear and transparent. These data indicate that ethyl azidoacetate reacts with the triphenylphosphine-based compound and triggers the disassembly of the coacervates by altering the hydrophobicity of the sticker. Taken together, we prove that the triphenylphosphine-PEG_8_-triphenylphosphine compound undergoes phase separation in the aqueous solution, and its reaction with ethyl azidoacetate disassembles the coacervates through the Staudinger reaction. This compound is, therefore, a Staudinger reaction-responsive phase-separating molecule, and the coacervates formed are called Staudinger Reaction-Responsive Coacervates (**SR-Coa**).

### **SR-Coa**-Mediated Intracellular Protein Delivery

**Figure 3:**
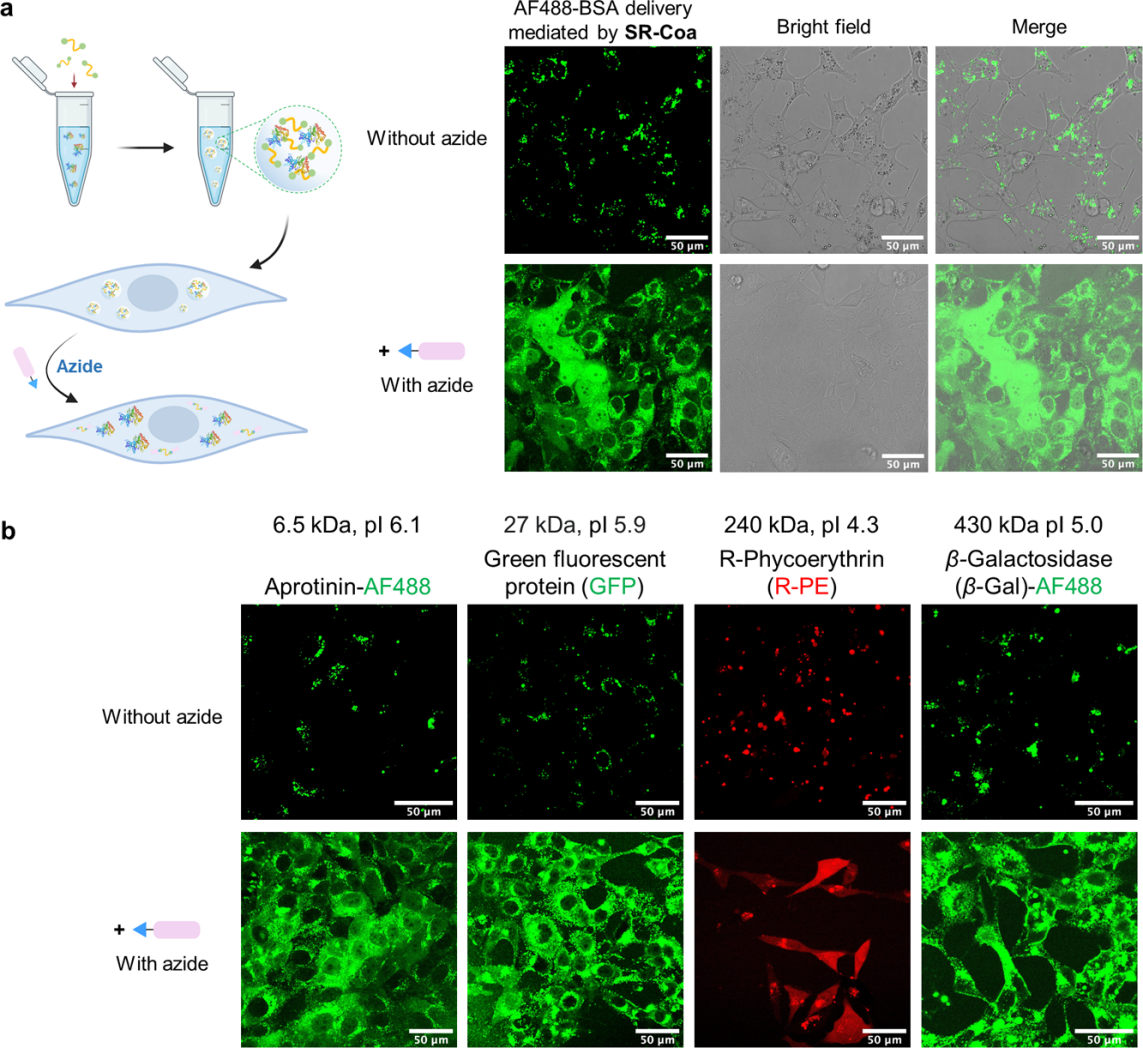
Cytosolic delivery of proteins by **SR-Coa**. (a) Confocal fluorescent microscopy images of HeLa cells treated with **SR-Coa** and AF488-BSA. Briefly, HeLa cells were incubated with **SR-Coa** at 0.1 mg/mL and AF488-BSA at 0.01 mg/mL, and ethyl azidoacetate (450 μM) was then added to the cell culture to disassemble the BSA-containing coacervates. (b) Confocal microscopic images showing that proteins with varying molecular weights and pI values could be delivered to the cytosol of HeLa cells by **SR-Coa**/ethyl azidoacetate treatment. β-Gal, β-galactosidase; R-PE, R-phycoerythrin; BSA, bovine serum albumin; GFP, green fluorescent protein. The fluorescence of Alexa Fluor 488, GFP, and R-PE was detected using the excitation/emission wavelengths of 488 nm/519 nm, 488 nm/507 nm, and 565 nm/575 nm, respectively.

Some coacervates are known to interact with and penetrate liposomes and cells; for example, in our previous work, PPFM coacervates could transport proteins into cells. Therefore, we next investigated the potential of **SR-Coa** to enter mammalian cells. After incubating **SR-Coa** with HeLa cells for 24 h, we observed Nile red-stained **SR-Coa** within the DiO-stained cell membrane (Figure S11). **SR-Coa** up to 0.3 mg/mL, ethyl azidoacetate up to 800 μM, or **SR-Coa** of 0.1 mg/mL plus ethyl azidoacetate of 800 μM did not induce an appreciable level of cytotoxic effects (Figures S12 and S13). Based on fluorescence-activated cell sorting (FACS) data, we observed a gradual but modest decrease in the fluorescent strength during the azidoacetate treatment, while the total number of fluorescent cells remained unchanged (Figure S14). This observation might reflect the azidoacetate-responsive redistribution of coacervate within the cytoplasm. Next, we loaded Alexa Fluor 488 (AF488)-labeled bovine serum albumin (BSA) at 0.01 mg/mL in **SR-Coa** (0.1 mg/mL) and incubated with HeLa cells for 24 h. Puncta of the fluorescent AF488 signal were found inside the cells in the fluorescent channel, which colocalized with **SR-Coa** puncta in the bright field. After adding ethyl azidoacetate (450 μM) to the **SR-Coa**-loaded cells, green puncta gradually dispersed during cell incubation, and the AF488 signal diffused throughout the cell cytosol (Figure 3a). These results show that **SR-Coa** can encapsulate proteins within the microdroplets and deliver them into the cells. The addition of ethyl azidoacetate caused the dispersion of the cargo protein into a wider space of the cytoplasm, presumably caused by the Staudinger reaction. Besides HeLa cells, the **SR-Coa**/ethyl azidoacetate system can also deliver proteins to other cell lines, including 293T, SK-BR-3, MDA-MB-231, and 3T3 (Figure S15).

Next, we show that aprotinin (with a molecular weight of 6.5 kDa and a pI of 6.1), GFP (27 kDa, pI 5.9), R-phycoerythrin (240 kDa, pI 4.3), and β-galactosidase (430 kDa, pI 5.0) can all be recruited at a concentration of 0.01 mg/mL in **SR-Coa** of 0.1 mg/mL. Protein-loaded coacervates effectively transported the selected proteins into HeLa cells, and ethyl azidoacetate treatment (450 μM) dispersed the cargo into the cytoplasm (Figures 3b and S16–S20). No colocalization of **SR-Coa**-delivered IgG and lysosomes was observed when we stained AF488-IgG/**SR-Coa**-treated HeLa cells with LysoTracker, similar to the photoresponsive coacervates we reported in a previous study^43^ (Figure S21). It is likely that such large liquid-like micron-scale droplets are uptaken by micropinocytosis, as demonstrated by Arafiles et al.^58^ and Shebanova et al.^59^ Next, we compared the delivery efficiency of **SR-Coa** with L17E (an endosomolytic cell-penetrating peptide)^32^ and PULSin (a commercial protein transfection reagent) using FACS analyses. PULSin delivery showed the highest efficiency for β-Gal (85.4%) and GFP (87.2%). The **SR-Coa** system gave the second highest efficiency (β-Gal (63.9%) and GFP (63.8%)), significantly higher than that of the L17E system (β-Gal, 3.9%; GFP, 20.1%) (Figure S22). Taken together, these data demonstrate that **SR-Coa** can deliver a wide range of proteins into the cytosol with an efficiency comparable to that of reported protein transfection reagents.

### Enzymatic Activity of SR-Coa-Delivered Peroxidase

**Figure 4:**
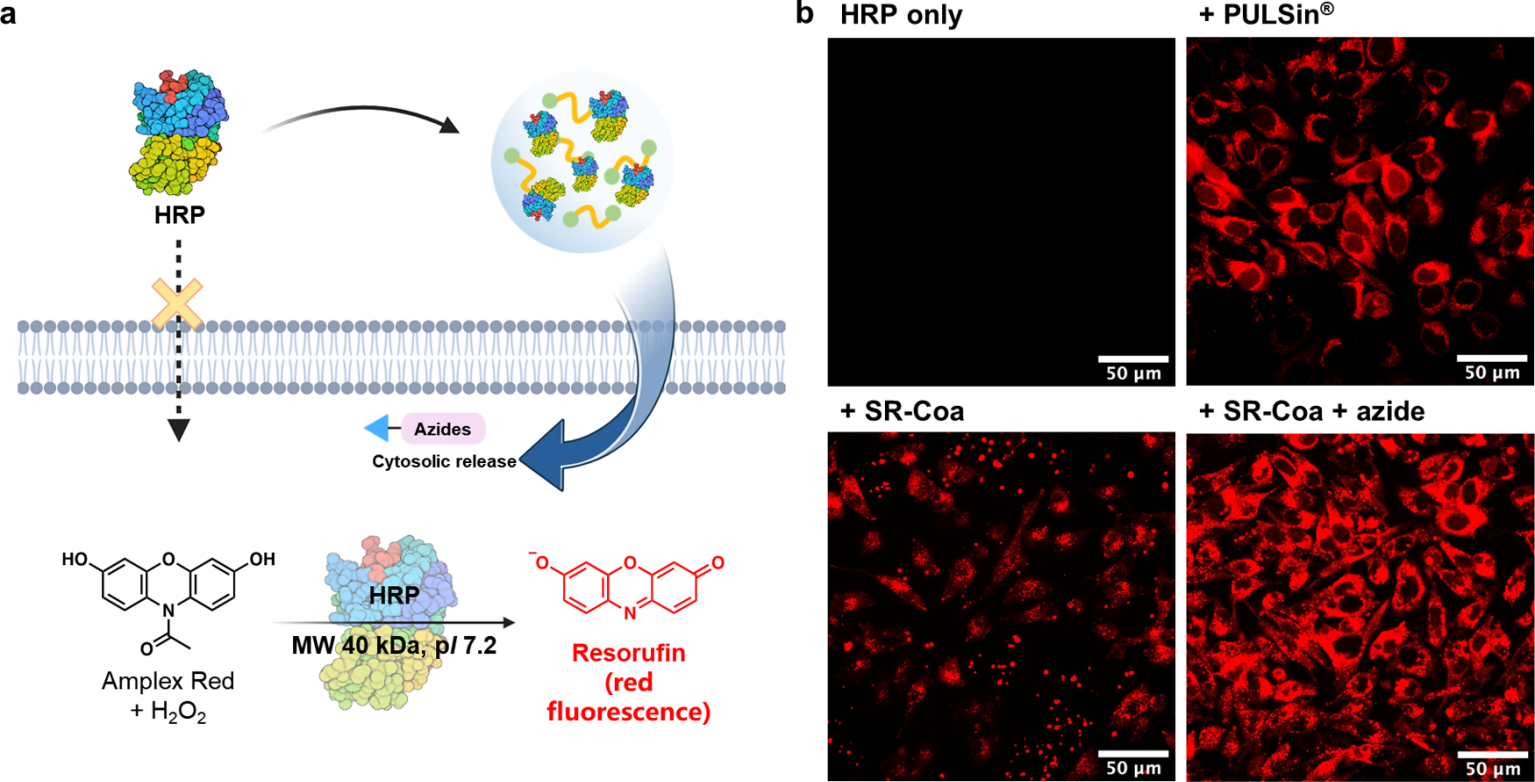
Intracellular activity of **SR-Coa**-delivered enzyme HRP. (a) Scheme showing HRP as a model for the coacervate-delivered enzyme in HeLa cells. (b) Confocal fluorescence microscopy images showing the distribution of resorufin, the enzymatic product of HRP, in the cell, following different treatments. The fluorescence of resorufin was detected using the excitation/emission wavelengths of 587 nm/610 nm.

Next, we prove that the **SR-Coa**-delivered enzyme retains its activity inside cells, using horseradish peroxidase (HRP) as a model. Amplex Red, a substrate for HRP, undergoes oxidation to resorufin (7-hydroxy-3*H*-phenoxazin-3-one), a red fluorescent compound, in the presence of hydrogen peroxide (H_2_O_2_) (Figure 4a). Upon entry of HRP-containing coacervates into the cell, ethyl azidoacetate was introduced to release HRP into the cytosol. Subsequent addition of Amplex Red and H_2_O_2_ resulted in red fluorescent puncta in the cell after 20 min. PULSin, a commercial protein-transfection reagent, was used as a positive control. Without **SR-Coa** or PULSin, no enzymatic reaction was observed inside the cells. Ethyl azidoacetate treatment caused a significantly more dispersed distribution of the fluorescent signal within the cytoplasm (Figures 4b and S23). Enzymatic catalysis was also observed using 3,3′,5,5′-tetramethylbenzidine (TMB) as the substrate, which turned blue after oxidation by HRP (Figure S24). These data demonstrate that **SR-Coa**-delivered HRP maintains enzymatic activity after being released into HeLa cells.

### Intracellular Delivery of an Anti-EZH2 Antibody

**Figure 5:**
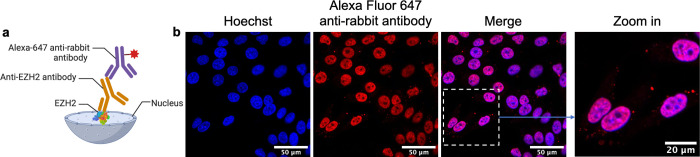
Nucleoplasm localization of an **SR-Coa**/ethyl azidoacetate-delivered anti-EZH2 antibody in live cells. (a) Schematic illustration showing the detection of EZH2 using an anti-EZH2 antibody. The primary antibody anti-EZH2 was introduced by the coacervate with cell permeabilization. (b) Immunofluorescent stained HeLa cells by the coacervate-delivered anti-EZH2 antibody. Briefly, HeLa cells were incubated with an anti-EZH2 antibody delivered by **SR-Coa** and treated with ethyl azidoacetate. The cells were fixed, permeabilized, blocked, and incubated with a fluorescent antirabbit antibody labeled with Alexa Fluor 647 to examine the intracellular location of the anti-EZH2 antibody. Alexa Fluor 647 dye and Hoechst 34580 were detected using the excitation/emission wavelengths of 647 nm/680 and 392 nm/440 nm, respectively.

Next, we examine whether **SR-Coa**/ethyl azidoacetate-delivered antibodies can recognize their targets inside the cells. We chose an antibody against enhancer of zeste homologue 2 (EZH2) as a model. EZH2 is a histone-lysine *N*-methyltransferase enzyme that participates in histone methylation. A subunit of a polycomb repressor complex, EZH2 is recurrently mutated in several forms of cancer and is highly expressed in numerous others, so inhibition of EZH2 has been considered a potential therapeutic option for various diseases.^60^ The subcellular location of EZH2 is the nucleoplasm. Regular immunofluorescent staining against nucleoplasm-located antigens requires fixation and permeabilization of the cells before incubation with the antibody. Here, we aim to omit the pretreatment step and deliver the antibody via the **SR-Coa**/ethyl azidoacetate strategy into live cells. Briefly, an anti-EZH2 antibody was mixed with **SR-Coa** and incubated with HeLa cells for intracellular delivery, followed by ethyl azidoacetate treatment to disperse the antibody into the cytosol. After washing the cells, we fixed, permeabilized, and incubated them with a secondary antibody against the EZH2 antibody, an Alexa Fluor 647-labeled antirabbit antibody (Figure 5a). Microscopic images of the cells showed that the fluorescent secondary antibody successfully labeled the nuclei, which were costained with a Hoechst dye (Figures 5b and S25). This result shows that the anti-EZH2 antibody delivered by the **SR-Coa**/ethyl azidoacetate system specifically targets the nuclei in live cells, where EZH2 is located. The same results were observed in other cell lines (Figures S26 and S27). In contrast, PULSin only delivered the anti-EZH2 antibody to the cytoplasm but failed to stain the nuclei (Figure S28), possibly due to endosomal entrapment.

### **SR-Coa**/Ethyl Azidoacetate-Mediated TRIM21-Dependent Protein Degradation

**Figure 6:**
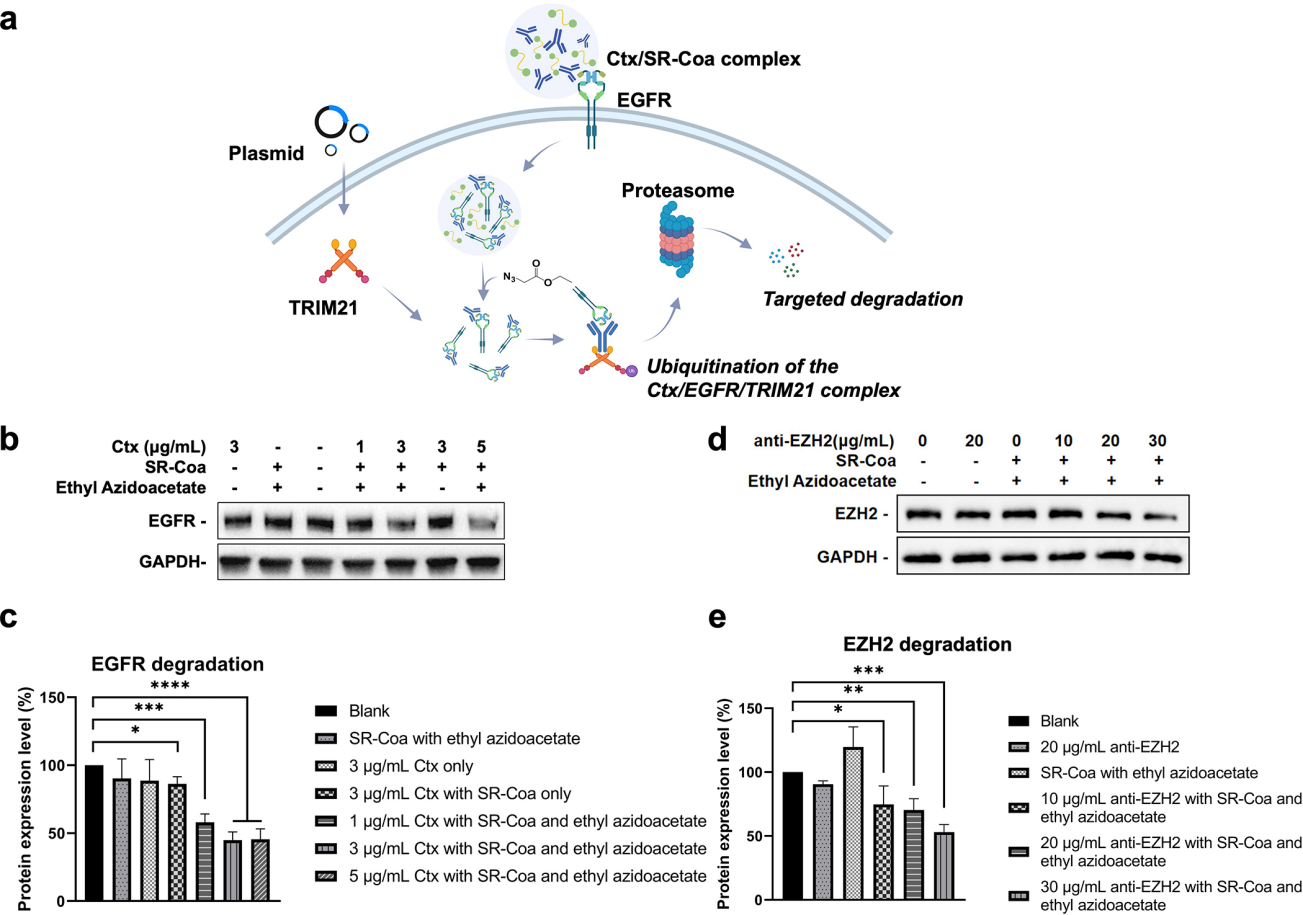
**SR-Coa**/ethyl azidoacetate-mediated TRIM21-dependent degradation of EGFR and EZH2. (a) Schematic illustration showing **SR-Coa**/ethyl azidoacetate-mediated Trim-Away for EGFR degradation. (b) Western blotting analysis of the EGFR expression level. (c) Statistical analysis of the EGFR expression level in different treatment groups. (d) Western blotting analysis of the EZH2 expression level. (e) Statistical analysis of EZH2 expression level in different treatment groups. Data are presented as the mean ± s.d. of *n* = 3 independent experiments. Statistical significance was calculated using the *t* test. *P < 0.05, ** *P* < 0.01, ***P < 0.001, ****P < 0.0001.

Trim-Away is a method for protein degradation based on the endogenous protein TRIM21 and the intracellular action of an exogenous antibody.^18^ TRIM21 binds the Fc domain of antibodies with a high affinity^61^ and recruits the antibody–antigen complex to the ubiquitin-proteasome system, leading to the degradation of the whole complex^62^ (Figure 6a). We overexpressed TRIM21 in SKBR3 cells to ensure a sufficient TRIM21 level. The cells were then incubated with **SR-Coa** encapsulating an anti-EGFR antibody, cetuximab (Ctx), followed by ethyl azidoacetate treatment. Western blotting showed that **SR-Coa** with 3 μg/mL or 5 μg/mL Ctx significantly reduced the EGFR level to about 45% of the original level in 12 h after ethyl azidoacetate treatment (Figure 6b,c). Without ethyl azidoacetate, Ctx/**SR-Coa** did not give appreciable EGFR reduction, similar to other control groups, including blank, **SR-Coa**/ethyl azidoacetate treatment only, and Ctx treatment only. These data suggest that **SR-Coa**/ethyl azidoacetate treatment facilitates the translocation of the EGFR/Ctx complex into cells, which was then recognized by intracellular TRIM21, an E3 ligase, for TRIM21-dependent degradation. Ethyl azidoacetate treatment was required for the EGFR/Ctx complex to bind TRIM21 inside the cell.

Next, we explored whether the **SR-Coa**/ethyl azidoacetate strategy could degrade EZH2, an intracellular protein. Briefly, HeLa cells overexpressing TRIM21 were incubated with **SR-Coa** packaged with an anti-EZH2 antibody, followed by ethyl azidoacetate treatment. Based on Western blotting, treatment of **SR-Coa** with 30 μg/mL anti-EZH2 antibody significantly reduced the EZH2 level to about 53% of the original level in 12 h (Figure 6d,e). Antibody alone without **SR-Coa** caused only a slight downregulation in protein expression, possibly through other unknown effects. Together, these data indicate that the **SR-Coa**/ethyl azidoacetate system can facilitate the targeted degradation of both EGFR and EZH2 via the TRIM21-dependent pathway.

Lastly, we examined whether the **SR-Coa**/ethyl azidoacetate system can degrade EGFR in vivo in a subcutaneous cancer model. Briefly, we inoculated SKBR3 cells subcutaneously into female BALB/c nude mice. After 21 days (set as day 0), the following solutions were injected into the tumor of mice: (I) recombinant TRIM21 protein (3 μg) with PBS (TRIM21/PBS group), (II) TRIM21 with anti-EGFR antibody Ctx (1.5 μg) (TRIM21/Ctx group), (III) TRIM21 with Ctx and **SR-Coa** (TRIM21/Ctx/**SR-Coa** group), and (IV) TRIM21 with Ctx and **SR-Coa**, subsequently followed by intratumoral injection of an ethyl azidoacetate solution (TRIM21/Ctx/**SR-Coa**/ethyl azide acetate group). After 12 h, the tumors were dissected and analyzed. In a pilot experiment, we intratumorally injected Nile red-labeled **SR-Coa** with or without the following ethyl azidoacetate injection. We observed that ethyl azidoacetate treatment significantly increased the distribution of Nile red-labeled **SR-Coa** in the tumor tissue (Figure S29a). Immunofluorescent staining of the four groups showed a significant reduction of the EGFR level in the TRIM21/Ctx/**SR-Coa** group compared with the TRIM21/Ctx group. In contrast, the TRIM21/Ctx/**SR-Coa**/ethyl azidoacetate group showed the lowest EGFR level (Figure S29b). These data indicate that **SR-Coa**-mediated delivery of the TRIM21/Ctx complex successfully degraded EGFR in the tumor, and intratumoral injection of ethyl azidoacetate further enhanced this effect. Hematoxylin/eosin (H&E) and TdT-mediated dUTP nick-end labeling (TUNEL) staining of the tissue sections showed a significantly higher level of apoptosis in the TRIM21/Ctx/**SR-Coa**/ethyl azidoacetate group, proving a potential tumoricidal effect in vivo (Figure S29c).

## Discussion

Changing the physical state of a molecule in vitro or in the cell may cause unexpected properties. A canonical example is aggregation-induced emission (AIE), a phenomenon in which molecules are weakly luminescent or nonluminescent in a dispersed state but have strong fluorescence in an aggregation state.^63^ Here, we present one class of triphenylphosphine-based compounds that can change their physical state from phase-separated coacervates to a solution triggered by a chemical reaction with the simple compound ethyl azidoacetate. The aggregated state, known as coacervates, can be used as a vehicle for intracellular protein translocation. Staudinger reaction of the coacervates with an azide dissolves the coacervates and releases the cargo protein to exert their biological activities toward intracellular targets. Therefore, this **SR-Coa**/ethyl azidoacetate system provides a solution for intracellular protein delivery. We use three examples to show that exogenous proteins delivered by the **SR-Coa**/ethyl azidoacetate system maintain their activity inside the cell: an enzyme can catalyze the chemical reaction, an antibody can recognize its target in the nuclei, and an antibody can guide its target to TRIM21-dependent protein degradation. Taken together, the control of the disassembly of coacervates, coupled with their ability to transport biomacromolecules across cell membranes, provides a powerful tool for protein and antibody delivery and targeted protein degradation and possibly a new cancer treatment.

## Supporting Information Available

Detailed experimental procedures, images of triphenylphosphine compounds with different linker lengths, characterization of the coacervates, encapsulation of different fluorescent dyes and proteins, data for screening azide compounds as coacervate disassembling agents, contact angles of the solutions, theoretical calculation data, peroxide-induced coacervate changes, kinetics of the click reaction, 3D microscopic images showing the location of coacervates in HeLa cells, cytotoxicity of the coacervates and ethyl azidoacetate, fluorescence-activated cell sorting of coacervate-treated cells, coacervate-mediated delivery of different proteins to different cells, comparison of different protein delivery agents, intracellular enzymatic activity data, nucleoplasm localization of the coacervate-delivered EZH2 antibody to different cell lines, coacervate-mediated EGFR degradation in vivo, and NMR spectra of all the compounds (PDF)
