# Supplementary material for: Staudinger Reaction-Responsive Coacervates for Cytosolic Antibody Delivery and TRIM21-Mediated Protein Degradation
Source: J Am Chem Soc. 2025 Jan 13;147(4):3830–9. doi: 10.1021/jacs.4c17054 (PMC11783599; doi:10.1021/jacs.4c17054)
Supplement: Supplementary file 1 — ja4c17054_si_001.pdf [file ja4c17054_si_001.pdf]

Supporting Information

Staudinger Reaction-Responsive Coacervates for Cytosolic Antibody Delivery and TRIM21-Mediated Protein Degradation

Yishu Bao,[a,†] Zhiyi Xu,[a,†] Kai Cheng,[*, a] Xiaojing Li,[a] Fangke Chen,[b] Dingdong Yuan,[a] Fang Zhang,[c] Audrey Run-Yu Che,[d] Xiangze Zeng,[b] Yuan-Di Zhao,[c] Jiang Xia[*, a]

[a] Department of Chemistry, The Chinese University of Hong Kong, Shatin, Hong Kong SAR, China.

[b] Department of Physics, Hong Kong Baptist University, Kowloon Tong, Hong Kong SAR, China.

[c] Britton Chance Center for Biomedical Photonics at Wuhan National Laboratory for Optoelectronics-Hubei Bioinformatics & Molecular Imaging Key Laboratory, Department of Biomedical Engineering, College of Life Science and Technology, Huazhong University of Science and Technology, Wuhan 430074, Hubei, P. R. China.

[d] Department of Natural Sciences, Pitzer and Scripps Colleges, 925 N. Mills Ave, Claremont, CA 91711.

*Address correspondence to kaicheng@cuhk.edu.hk, and jiangxia@cuhk.edu.hk ORCID 0000-0001-8112-7625

Phone: (852) 3943 6165
Fax: (852) 2603 5057

† Y. B. and Z. X. contributed equally.

Contents

Item	Page No.
**Detailed experimental procedures**	**S4**
**Figure S1.** The length of PEG linker affects the physical states of triphenylphosphine compounds.	**S16**
**Figure S2.** Physical properties of the **SR-Coa** coacervates.	**S17**
**Figure S3.** Confocal images of **SR-Coa** with different fluorescent dyes	**S18**
**Figure S4.** Quantification of recruitment efficiency.	**S19**
**Figure S5.** Screening azide compounds for coacervate disassembling agents.	**S20**
**Figure S6.** Contact angles of the triphenylphosphine compound solution measured before (a) and after Staudinger reaction (b).	**S21**
**Figure S7.** Hydration-free energies for model compounds mimicking the stickers.	**S22**
**Figure S8.** Treating **SR-Coa** with different concentrations of hydrogen peroxides.	**S23**
**Figure S9.** Quantification of the products during the reaction.	**S24**
**Figure S10.** NMR spectra of **6e**.	**S25**
**Figure S11.** 3D confocal microscopic images of HeLa cells treated with **SR-Coa** coacervates.	**S26**
**Figure S12.** Cytotoxicity of **SR-Coa** by the CCK8 assay.	**S27**
**Figure S13.** Cytotoxicity of the ethyl azidoacetate by the CCK8 assay.	**S28**
**Figure S14.** Fluorescence-activated cell sorting (FACS) study of azidoacetate responsiveness of **SR-Coa**-treated cells.	**S29**
**Figure S15.** Intracellular delivery of AF488-BSA to different cell lines.	**S30**
**Figure S16.** Intracellular delivery of AF488-aprotinin.	**S31**
**Figure S17.** Intracellular delivery of GFP.	**S32**
**Figure S18.** Intracellular delivery of R-PE.	**S33**
**Figure S19.** Intracellular delivery of AF488-β-Gal.	**S34**

**Figure S20.** Fluorescent proteins alone did not enter cells without **SR-Coa** coacervates.	**S35**
**Figure S21.** Confocal fluorescent microscopy images for cells incubated with AF488-IgG/**SR-Coa** and LysoTrackers.	**S36**
**Figure S22.** Comparison of the efficiency of protein delivery by different vehicles into HeLa cells.	**S37**
**Figure S23.** Intracellular HRP enzymatic activity.	**S38**
**Figure S24.** Intracellular HRP enzymatic activity analysis by TMB assay.	**S39**
**Figure S25.** Nucleoplasm localization of a coacervate-delivered anti-EZH2 antibody without permeabilization.	**S40**
**Figure S26.** Delivery of anti-EZH2 antibody into HEK 293T, SKBR3, MDA-MB-231, and 3T3 cell lines by **SR-Coa**.	**S41**
**Figure S27.** Anti-EHZ2 antibody alone did not enter 293T, SK-BR-3, MDA-MB-231, and 3T3 cell lines without **SR-Coa**.	**S42**
**Figure S28.** Delivery of anti-EZH2 antibody into HEK 293T by PULsin®.	**S43**
**Figure S29.** Coacervate-mediated EGFR degradation in *vivo*.	**S44**
**Characterization Data**	**S46**
**Figure S30.** NMR spectra of **6a**.	**S49**
**Figure S31.** NMR spectra of **6b**.	**S50**
**Figure S32.** NMR spectra of **6c**.	**S51**
**Figure S33.** NMR spectra of **6d**.	**S52**
**References**	**S53**

Detailed Experimental Procedures

Materials and Instruments

Unless otherwise specified, all reagents and solvents were purchased from commercial sources and were used without further purification. 1-Methyl 2-aminoterephthalate, palladium diacetate, and diphenylphosphine were purchased from Sigma-Aldrich Co. (MO, USA). Cytotoxicity assay kits were purchased from MedChemExpress (NJ, USA). Bovine Serum Albumin (BSA), Horseradish Peroxidase (HRP), R-Phycoerythrin (R-PE), and β-Gal were purchased from Thermo Fisher Scientific Inc. (MA, USA). The anti-EZH2 antibodies were purchased from Abcam Limited (UK). Dulbecco's modified Eagle medium (DMEM), fetal bovine serum (FBS), phosphate-buffered saline (PBS), and Penicillin-Streptomycin-Glutamine (100×) liquid were purchased from Thermo Fisher Scientific Inc.

Reactions were monitored through thin layer chromatography (TLC) on 0.30 mm SiliCycle silica gel plates and visualized under UV light. All known compounds were identified by ^{1}H NMR, ^{13}C NMR and compared with previously reported data. NMR spectra of the products were recorded using Bruker 500 MHz spectrometer, with tetramethylsilane as an internal reference. Chemical shifts (δ) and coupling constants (J) were expressed in ppm and Hz, respectively. The following abbreviations indicated the multiplicities: s, singlet; d, doublet; t, triplet; q, quartet; m, multiplet. High-resolution mass spectra (HRMS) were obtained on a Thermo Scientific Q Exactive Focus Mass Spectrometer with electron spray ionization (ESI).

General procedure (I) for the synthesis of compounds 4. [1]

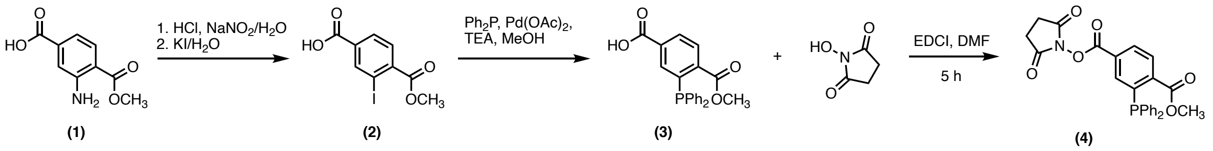

A solution of $NaNO_2$ (180 mg, 2.64 mmol) in 1 mL of H_2O was added dropwise to a solution of 1-methyl-2-aminoterephthalate **1** (500 mg, 2.56 mmol) in 5 mL of cold concentrated HCl. The mixture was stirred for 30 min at room temperature and then filtered into a solution of KI (4.30 g, 25.0 mmol) in 7 mL of H_2O. The dark red solution was stirred for 1 hour and then diluted with CH_2Cl_2 (100 mL) and washed with saturated Na_2SO_3 (2 × 10 mL). The organic layer was washed with water (2 × 20 mL) and saturated NaCl (1 × 20 mL). The combined aqueous layers were back extracted with CH_2Cl_2 (20 mL). The combined organic layers were dried over Na_2SO_4 and concentrated. The crude product was dissolved in a minimum amount of MeOH, and H_2O was added until the solution appeared slightly cloudy. Cooling the solution to 4°C and subsequent filtration gave a yellow solid **2**.

Compound **2** (300 mg, 1.00 mmol), dry MeOH (3 mL), triethylamine (0.3 mL, 2 mmol), and palladium acetate (2.2 mg, 0.010 mmol) were added to a flame-dried flask. While stirring under an atmosphere of Ar, diphenylphosphine (0.17 mL, 1.0 mmol) was added to the flask using a syringe. The resulting solution was heated at reflux for 12 hours, cooled to room temperature, and concentrated. The residue was dissolved in 250 mL of a 1:1 mixture of CH_2Cl_2/H_2O, and the layers were separated. The organic layer was washed with 1 M HCl (1 × 10 mL) and concentrated. The crude product was dissolved in a minimum amount of MeOH and an equal amount of H_2O was added. The solution was cooled to 4°C and the resulting solid was collected by filtration to give compound **3**.

To a solution of **3** (182.2 mg, 0.50 mmol) in DMF (3.0 mL) 1-hydroxypyrrolidine-2,5-dione (575.4 mg, 5 mmol), 1-ethyl-3-(3-dimethylaminopropyl) carbodiimide (EDCI) (388.1 mg, 2.5 mmol) were added. The resulting mixture was stirred with a magnetic stirrer at room temperature under air for 5 h. The reaction solution was washed with water (3 × 30 mL). The combined organic layers were dried over Na_2SO_4 and concentrated. The residue was purified by flash column chromatography on silica gel (DCM) to give **4**.

General procedure (II) for the synthesis of triaryl phosphine-based molecules 6

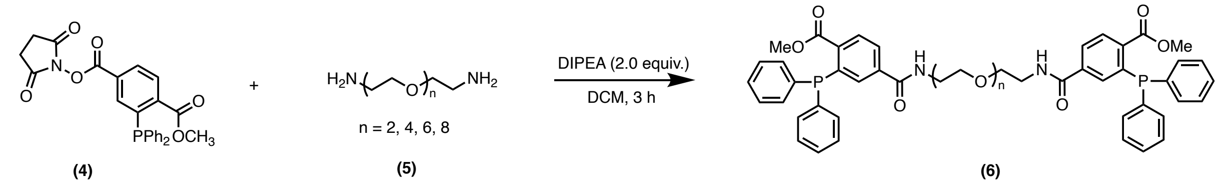

To a solution of **4** (92.3 mg, 0.20 mmol) in DCM (2.0 mL) amine-PEG$_n$-amine **5** (0.10 mmol) and DIPEA (25.8 mg, 0.20 mmol) were added. The resulting mixture was stirred with a magnetic stirrer at room temperature under air for 3 h. Then, the reaction mixture was concentrated in vacuo. The residue was purified by flash column chromatography on silica gel (MeOH: DCM = 1: 100) to give **6**.

Preparation of coacervates

Triarylphosphine-based molecules were dissolved in DMSO (100 mg/mL, stock solution), and then 10 μL stock solution was added to 90 μL aqueous solution gently to allow the coacervates to form. The solution immediately became white and milky, indicating the formation of coacervates. Coacervates were visualized under a confocal microscope (Stellaris 8, Leica, Wetzlar, Germany).

Cell culture

All cell lines were cultured in a humidified atmosphere (at 37 °C, 5% CO_2), and maintained in Dulbecco's modified eagle's medium (DMEM, Gibco, Thermo Fisher) supplemented with 10% fetal bovine serum (FBS, Gibco, Thermo Fisher) and Penicillin-Streptomycin-Glutamine (100×, Gibco, Thermo Fisher) before the addition of protein-containing solutions. SKBR3 cells, an epidermal growth factor receptor (EGFR) highly expressed human breast cancer cell line, were cultured in McCoy's 5A medium, supplemented with 10% fetal bovine serum. Cells were maintained in 5% CO2 atmosphere at 37°C.

Plasmids construction

Gene encoding TRIM21 was amplified from the cDNA of HeLa cells with forward primer ACTATAGGGAGAGCCATGGCTTCAGCAGCAC and reverse primer GGTGATGATGACCGGaATAGTCAGTGGATCCTTGTGA. The pCMV backbone was amplified with forward primer CCGGTCATCATCACCATCA and reverse primer GGCTCTCCCTATAGTGAGT. The TRIM21 gene and pCMV backbone were cloned by ClonExpress UItra One Step Cloning Kit (C115, Vazyme). Recombination products were transformed into DH5α competent cells and screened with ampicillin to get correct pCMV-TRIM21 plasmids.

Cytotoxicity assay by Cell Counting Kit-8 (CCK-8)

100 μL of HeLa cell suspension (5000 cells/ well) was seeded in a 96-well plate (Thermo Scientific), and pre-incubated for 24 h in a humidified incubator (at 37 °C, 5% CO_2). Cells were washed with PBS

once before adding 100 uL serum-free media. Then 10 μL of coacervates of various concentrations was added into the culture media in the plate. After incubating for 4 hours, the medium was removed, and the cells were washed twice with PBS buffer. Then, 100 μL fresh medium (DMEM, 10% FBS, Penicillin-Streptomycin-Glutamine) was added. The cells were incubated for another 20 hours. After that, 10 μL of CCK8 solution was added to each well of the plate. Special care was taken to avoid bubbles in the wells since they interfere with the O.D. reading. After 1 hour in the incubator, the absorbance at 450 nm was measured using a microplate reader.

Cellular entry of SR-Coa coacervates

For cellular delivery, 3×10^4 cells were suspended in 1 mL of DMEM supplemented with 10% FBS and Penicillin-Streptomycin-Glutamine and then transferred into 20 mm confocal dishes (NEST Scientific, Wuxi, China). After incubating for 24 hours, the medium was replaced with the coacervate-containing solution (DMEM, without FBS, 0.1 mg/mL **SR-Coa** compound). After incubation for 4 hours, the medium was removed, and the cells were washed twice with PBS buffer. Then, 1 mL fresh medium (DMEM, 10% FBS, Penicillin-Streptomycin-Glutamine) was added. The cells were incubated for another 20 hours and then washed with PBS twice before they were imaged under a confocal microscope (Stellaris 8, Leica, Wetzlar, Germany).

Measurement of turbidity and encapsulation/recruitment of different dyes/proteins in the coacervates

Phase separation of the triphenylphosphine-based compounds was monitored according to the turbidity of the solution using a microplate reader (Thermo Scientific™, A51119600C, USA). Briefly, the fluorescence signal of fluorescent molecule solutions (dyes: 0.02 mM, proteins: 0.01 mg/mL) was measured as F0. After 1 μL of **SR-Coa** compound (250 mg/mL) was added to the fluorescent solution (49 μL) to allow the formation of dye-encapsulated coacervates, the solution was centrifuged, and the fluorescence signal of the supernatant was measured as F1. The recruitment efficiency was calculated as (F0-F1)/F0×100%. [2] Data are presented as the mean ± s.d. of at least 3 independent experiments. The fluorescence of RhB, Rh6G, SYBR Green, Nile red, Kiton Red, Cy3, 5-FAM, Alexa Fluor™ 488 (AF488), GFP, and R-PE was detected by a microplate reader (Thermo Scientific™, A51119600C, USA) using the excitation/emission wavelengths of 555 nm / 580 nm, 530 nm / 556 nm, 497 nm / 520 nm, 549 nm / 628 nm, 556 nm / 575 nm, 550 nm / 570 nm, 495 nm / 520 nm, 488 nm / 519 nm, 488 nm / 507 nm, and 565 nm / 575 nm, respectively.

SR-Coa-mediated protein delivery

Alexa Fluor™ 488-labeled proteins (including aprotinin, BSA, GFP, R-PE, *β*-Gal) were stocked in PBS buffer at the concentration of 1 mg/mL. For cell culture experiments, the stock solution (100 mg/mL **SR-Coa** compound in DMSO) was mixed with the cargo protein in PBS in a 1:10 ratio (protein : vehicle) to give a coacervate solution containing 10 mg/mL **SR-Coa** compound and 1 mg/mL cargo protein with a DMSO/water ratio of 1:10. Before cells were added, the coacervate-containing solution was further diluted in 1:100 ratio in DMEM without FBS to a final concentration of 0.01 mg/mL protein and 0.1 mg/mL **SR-Coa** compound.

For protein delivery into cells, 3×10^4 cells were suspended in 1 mL of DMEM (10% FBS and Penicillin-Streptomycin-Glutamine) and then transferred into 20 mm confocal dishes (NEST Scientific). After 24 h of incubation, the medium was replaced with protein-containing coacervates (DMEM, without FBS, with Penicillin-Streptomycin-Glutamine, 0.01 mg/mL protein, and 0.1 mg/mL **SR-Coa** coacervates). After incubating for 4 hours, the medium was removed, and the cells were washed twice with PBS buffer. Then, 1 mL fresh medium (DMEM, 10% FBS, Penicillin-Streptomycin-Glutamine) was added. The cells were incubated for another 20 hours. Then, the medium was removed, and 1 mL ethyl azideacetate (450 μM)-containing fresh medium was added and incubated for another 12 hours. Cells were washed with PBS twice before they were imaged under a confocal microscope (Stellaris 8, Leica). The same protocol was used for other mentioned cell lines. The fluorescence of Alexa Fluor™ 488, GFP, and R-PE was detected using the excitation/emission wavelengths of 488 nm / 519 nm, 488 nm / 507 nm, and 565 nm / 575 nm, respectively.

Study on internalization mechanism

Cells were stained with LysoTracker™ Red DND-99 (Invitrogen, Thermo Fisher) by the manuals from the manufacturer. The total process is protected from light. Before staining, the HeLa cell was treated with AF488 IgG-containing SR-Coa using the methods mentioned above. Then, cells were stained with Hoechst 34580 PBS solution (8.1 μM) for 10 minutes. Then, the treated HeLa cells were washed with PBS twice before imaging. The fluorescence of LysoTracker, AF488 IgG, and Hoechst 34580

was detected using excitation/emission wavelengths of 577 nm / 590 nm, 488 nm / 519 nm, and 392 nm / 440 nm, respectively.

HRP activity assay

After delivery of HRP into HeLa cells by the coacervates, cells were treated with ethyl azideacetate and then incubated for another 12 hours. After being washed three times with PBS buffer, the cells were incubated in Amplex Red (50 μM) and hydrogen peroxide (500 μM) in the PBS buffer. After incubation for 30 min at room temperature, the cells were washed with PBS buffer three times and observed under a confocal microscope (Stellaris 8, Leica). In another typical intracellular enzyme activity assay, cytosolic HRP activity was analyzed using a similar procedure but with a TMB substrate. We dissolved TMB substrate in 3 mM hydrogen peroxide containing acetate buffer (pH 5) and added TMB-containing buffer to each well. Cells were added to each well and incubated for 10 minutes at room temperature before observation under the microscope. The fluorescence of Resorufin was detected using the excitation and emission wavelengths of 570 nm and 585 nm.

Delivery of the anti-EZH2 antibody

An anti-EZH2 antibody (ab245738 from Abcam, UK, 1 μg/μL) was mixed with **SR-Coa** and delivered into HeLa cells. The same protocol was used for other mentioned cell lines. Ethyl azidoacetate was then added to the cells. Then, the cells were fixed using 4% paraformaldehyde in PBS pH 7.4 for 15 minutes at room temperature. The cells were washed three times with PBS. Then, we incubated the cells for 10 min with PBS containing 0.2% Triton X-100. After extensive washing, we incubated cells

with 1% BSA (22.52 mg/mL) in PBST (PBS + 0.1% Tween 20) for 30 min to block the nonspecific binding of the antibodies. Next, cells were incubated with an Alexa-647 anti-rabbit secondary antibody in 1% BSA for 3 hours at room temperature in the dark. The fluorescence of Alexa Fluor 647 anti-rabbit antibody and Hoechst 34580 was detected using the excitation/emission wavelengths of 647 nm / 680 nm and 392 nm / 440 nm, respectively.

Coacervates-mediated TRIM-AWAY

EGFR TRIM-AWAY: SKBR3 cells were firstly transfected with the pCMV-hTRIM21 and incubated for 24 h. Then, cells were digested with trypsin and re-seeded in a 24-well plate at a density of 60%. After 7 h, 0 μg, 0.5 μg, 1.5 μg, and 2.5 μg of anti-EGFR antibody, Cetuximab (Ctx), were added into 5 μL Opti-MEM medium. After that, 0.5 μL of 100 mg/mL SR-Coa stock solution was mixed with the former medium. After 10 min incubation, 500 μL fresh Opti-MEM medium was added to each tube. This antibody/coacervate-containing medium was used to replace the old cell medium and incubate with cells for 4 hours. Then, the medium was replaced by the fresh complete medium for 20 hours, and the ethyl azidoacetate (5 eq.) was added to the medium for 12 hours. The cells were collected for Western Blotting analysis afterward.

EZH2 TRIM-AWAY: HeLa cells were firstly transfected with the pCMV-hTRIM21 and incubated for 24 h. Then cells were digested with trypsin and re-seeded in a 24-well plate at a density of 60%. After 7 h, 0 μg, 5 μg, 10 μg, and 15 μg EZH2/KMT6 rabbit mAb (A19577, Abclonal) were added into 5 μL Opti-MEM medium. After that, 0.5 μL of 100 mg/mL RPMs stock solution was mixed with the former medium. After 10 min incubation, 500 μL fresh Opti-MEM medium was added into each tube.

This antibody/coacervate-containing medium was used to replace the old cell medium and incubate with cells for 4 hours. Then, the medium was replaced by the fresh complete medium for 20 hours, and the ethyl azidoacetate (5 eq.) was added to the medium for 12 hours. The cells were collected for Western Blotting analysis afterward.

Western blotting for EGFR and EZH2

Glyceraldehyde-3-phosphate dehydrogenase (GAPDH) was used as the internal control. Collected cells were washed with PBS and lysed with RIPA buffer. The protein concentrations were detected by BCA assay. 15 μg of protein were taken from each sample for analysis. Samples were mixed with 6× loading dye and incubated at 50 °C for 10 min before loaded to the 10% SDS-PAGE gel. After gel electrophoresis, proteins on the gel were transferred to polyvinylidene fluoride membranes (Immobilon-P, Millipore). Membranes were blocked in PBST-containing 5% skim milk powder (Blotting grade, Beyotime) for 1 hour at 25 °C and incubated with primary antibody for 12 hours at 4 °C. The primary antibodies of rabbit anti-GAPDH (AC027, 1:5000, Abclonal), mouse EGFR monoclonal antibody (66455-1-Ig, 1:5000, Proteintech) and mouse EZH2 monoclonal antibody (66476-1-Ig, 1:10000, Proteintech) were diluted in PBST solution, respectively. The goat anti-rabbit HRP-conjugated secondary antibodies (RGAR001, 1:10000, Proteintech) and goat anti-mouse HRP-conjugated secondary antibodies (7076, 1:2000, Cell Signaling Technology) were diluted in PBST solution, respectively. Membranes were incubated with secondary antibodies for 1 hour at 25 °C. The membrane was detected with the Ultra High Sensitivity ECL Kit (HY-K1005, MedChemExpress) by

ChemiDoc^{TM} Touch Imaging System (1708370, BIO-RAD). Image J was used to quantify the Western Blot data.

Force field parameters for small organic molecules

We used the second generation of General Amber Force Field (GAFF2) parameters [3] for all-atom molecular dynamics simulations of small organic molecules. The antechamber Python parser interface (ACPYPE) was used to derive the bonded and non-bonded interaction parameters [4]. The partial charge was further optimized by fitting to a HF/6-31G* level electric potential on an online RESP ESP charge Derive (R.E.D.) Server [5]. The restrained electrostatic potential (RESP) fitting method was used [6]. The transferable intermolecular potential 3P (TIP3P) water model was used [7].

Hydration free energy calculation using all-atom molecular dynamics simulations

All the simulations were performed using GROMACS 2023 package [8]. The Bennett Acceptance Ratio (BAR) method was used to obtain the hydration free energies of organic molecules of interest [9]. We used a list of λ vectors to scale the Van Der Waals and Coulomb electrostatic interaction strengths between the organic molecule and waters: [λ_{vdw}, λ_{coul}] = [0.00, 0.00], [0.10, 0.00], [0.20, 0.00], [0.30, 0.00], [0.40, 0.00], [0.50, 0.00], [0.60, 0.00], [0.70, 0.00], [0.80, 0.00], [0.90, 0.00], [1.00, 0.00], [1.00, 0.10], [1.00, 0.20], [1.00, 0.30], [1.00, 0.40], [1.00, 0.50], [1.00, 0.60], [1.00, 0.70], [1.00, 0.80], [1.00, 0.90], [1.00, 1.00]. For each λ vector, the organic molecule was solvated in a $5 \times 5 \times 5\ nm^3$ water box followed by 10 ns NPT simulations at 310K and 1 bar. The V-rescale

method [10] and Perriello-Rahman method [11] were used to maintain the temperature and pressure, respectively. The temperature coupling constant was 0.1 ps and the pressure coupling constant was 2.0 ps. Then we used *gmx bar* module in GROMACS to calculate the free energy difference between two adjacent states and obtained the hydration free energy for each organic molecule.

Coacervates-mediated antitumor therapy in *vivo*

About 1×10^6 SKBR-3 cells were inoculated subcutaneously in 5-week-old BALB/c female nude mice. After 21 days, they were randomly divided into 5 groups with 5 mice per group, and 50 μL of different formulations (I)PBS, (II) SR-Coa with ethyl azidoacetate, (III) Ctx, (IV) SR-Coa+Ctx, (V)SR-Coa+Ctx with ethyl azidoacetate were injected intratumorally (SR-Coa: 0.3 mg/mL; Ctx: 0.03 mg/mL; ethyl azidoacetate (1.73 mM): 20 μL; 3 μg of TRIM21 proteins were delivered to each group simultaneously, and the PBS and Ctx group were directly mixed). Group V was injected again 24 hours later. After another 12 hours, one mouse in each group was euthanized for apoptosis analysis of DAPI, hematoxylin/eosin (H&E) and TdT-mediated dUTP Nick-End Labeling (TUNEL) staining analysis, and EGFR immunofluorescence (secondary antibodies: FITC-labeled Goat Anti-Rabbit IgG), then the results were observed under the confocal microscope. On day 8, tumors are collected for weight and volume analysis. Nile red, FITC, and DAPI were detected using the excitation/emission wavelengths of 549 nm / 628 nm, 488 nm / 519 nm, and 364 nm / 454 nm, respectively.

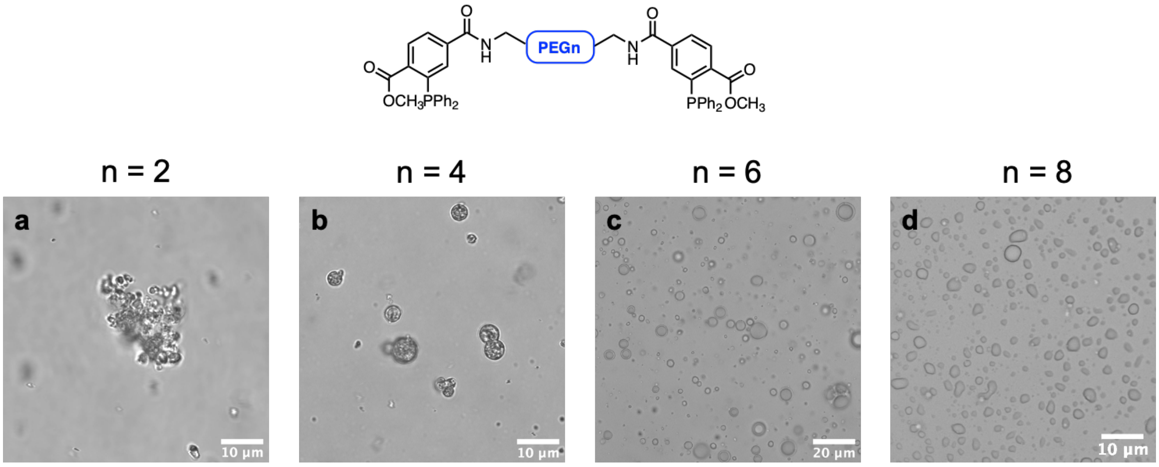

Figure S1. The length of PEG linker affects the physical states of triphenylphosphine compounds.

a, Aggregates. **b,** Uneven droplets/precipitates. **c,** Hollow structures. **d,** Coacervate microdroplets.

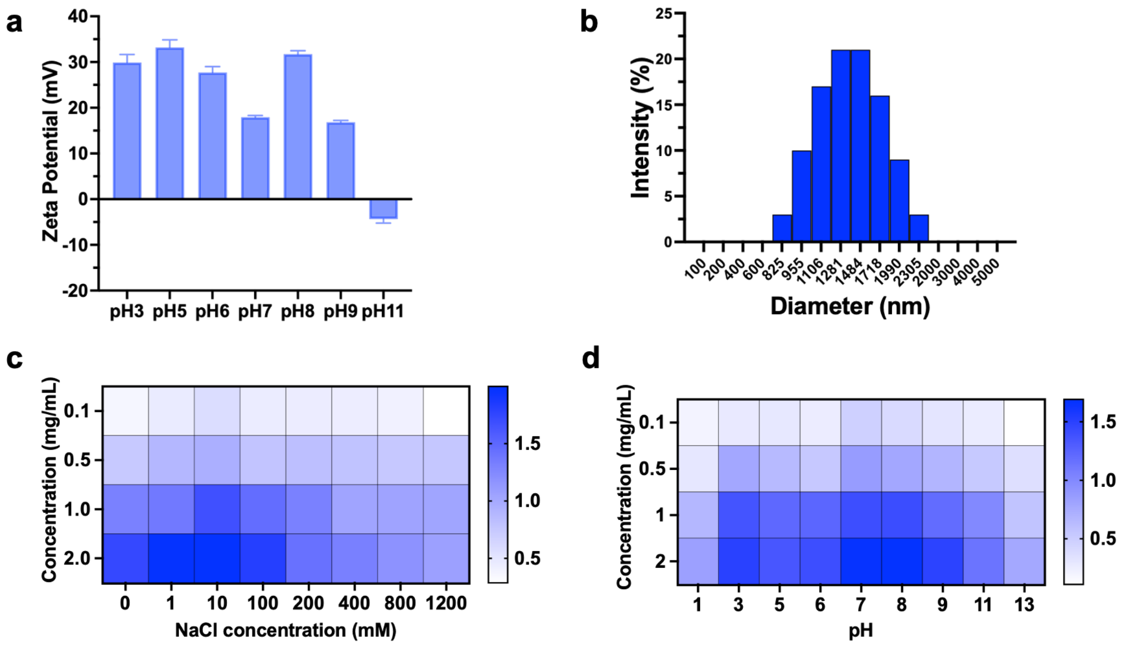

Figure S2. Physical properties of the SR-Coa coacervates. **a**, Zeta potential of **SR-Coa** coacervates at 0.1 mg/mL and different pH values. **b**, The size of coacervates measured by dynamic light scattering (DLS) at 0.1 mg/mL concentration (pH 7, 100 mM NaCl). **c**, Turbidity of **SR-Coa** coacervates at different concentrations and ionic strengths. **d**, Turbidity of **SR-Coa** coacervates at different pH values and concentrations.

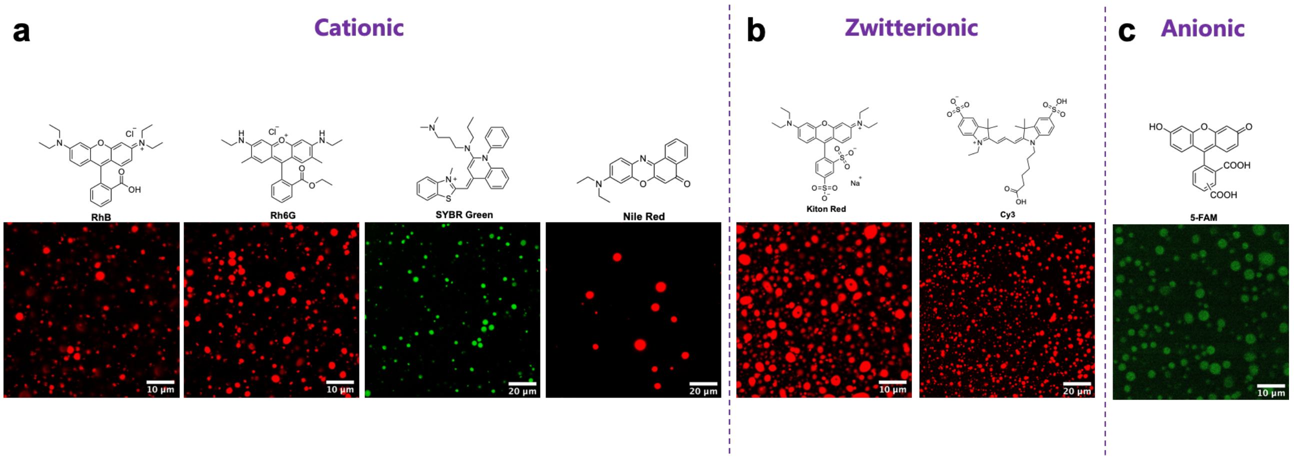

Figure S3. Confocal images of SR-Coa with different fluorescent dyes. a, Cationic dyes. The fluorescence of RhB, Rh6G, SYBR Green, and Nile red was detected using the excitation/emission wavelengths of 555 nm / 580 nm, 530 nm / 556 nm, 497 nm / 520 nm, and 549 nm / 628 nm, respectively. **b, Zwitterionic dyes.** The fluorescence of Kiton Red and Cy3 was detected using the excitation/emission wavelengths of 556nm / 575nm and 550nm / 570nm, respectively. **c, Anionic dye.** The fluorescence of 5-FAM was detected using the excitation/emission wavelengths of 495 nm / 520 nm. The concentration of fluorescent dyes was 0.02 mM and **SR-Coa** compound was 4.52 mM.

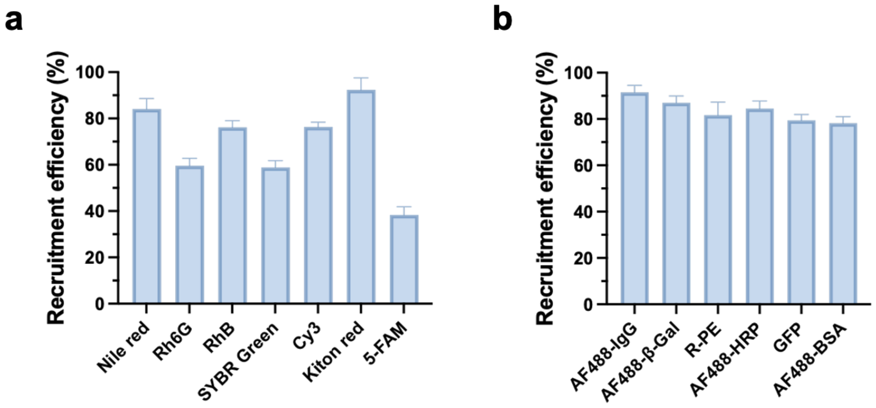

Figure S4. Quantification of recruitment efficiency. a, dyes; b, proteins. Briefly, the fluorescence signal of the dyes/proteins (dyes: 0.02 mM, proteins: 5 μg/mL) was measured as F0. **SR-Coa** was added to the dyes/protein solution to a final molecular concentration of 4.52 mM (0.05 mg/mL). The solution was then centrifuged, and the fluorescence signal of the supernatant was measured as F1. The recruitment efficiency was calculated as (F0-F1)/F0×100%. Data are presented as the mean ± s.d. of n = 3 independent experiments.

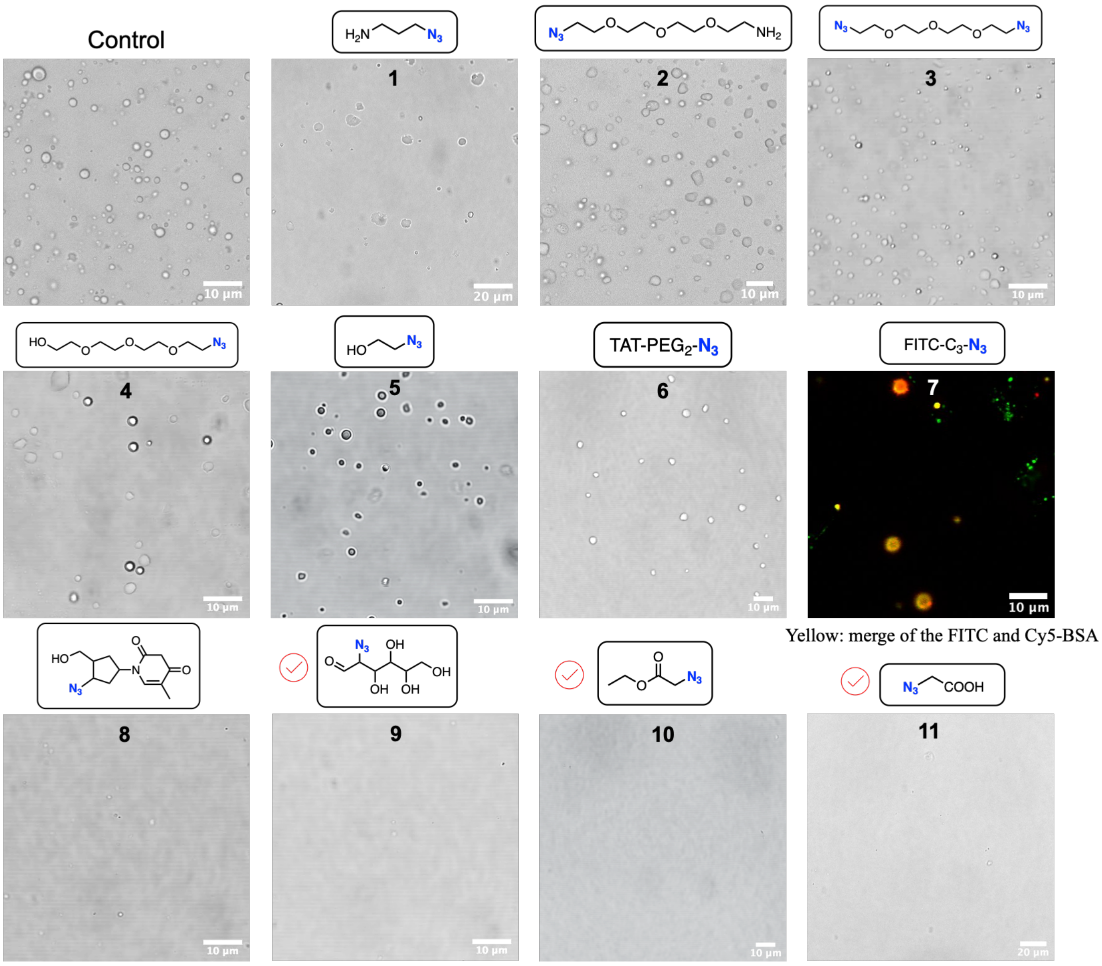

Figure S5. Screening azide compounds for coacervate disassembling agents. Briefly, coacervate solutions were prepared in the PBS buffer, then different azides-containing compounds (5 equiv.) were added and incubated at 37 °C for 2 hours before the reaction solutions were observed under microscopy. Entries 9-11 show complete dissolution of the coacervates.

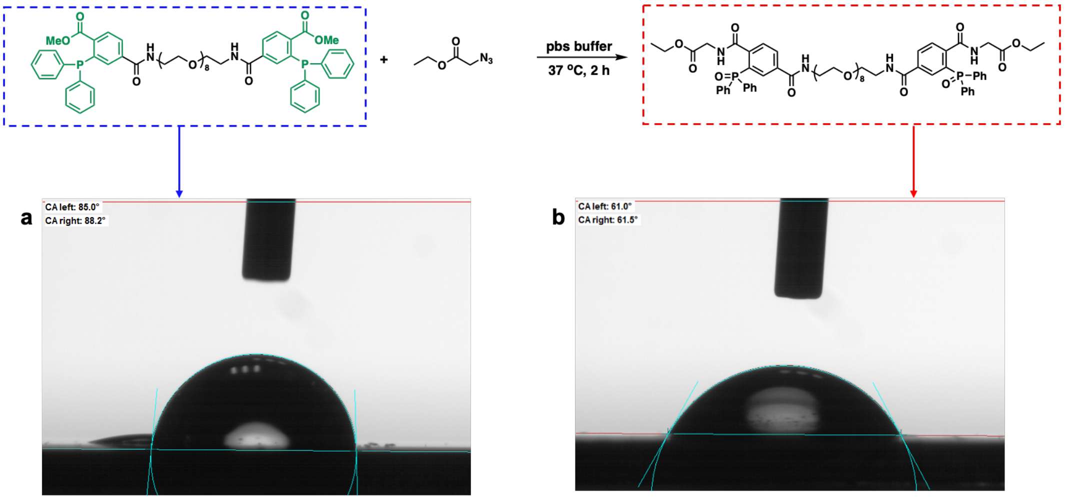

Figure S6. Contact angles of the triphenylphosphine compound solution measured before (a) and after Staudinger reaction (b). Smaller contact angles mean higher hydrophilicity. Therefore, the product is more hydrophilic than the reactant.

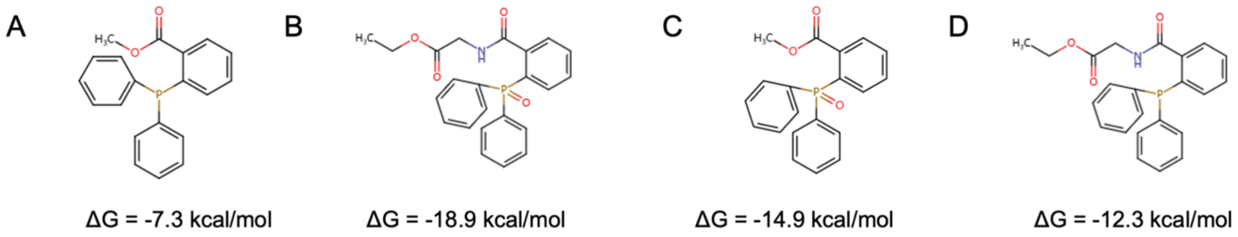

Figure S7. Hydration-free energies for model compounds mimicking the stickers. (A) triarylphosphine, **(B)** triarylphosphine oxide with the amide group, **(C)** triarylphosphine oxide without the amide group, and **(D)** triarylphosphine with the amide group. The decrease of hydration free energy after the reaction can be attributed to the oxidation of the phosphate atom and the addition of one amide group.

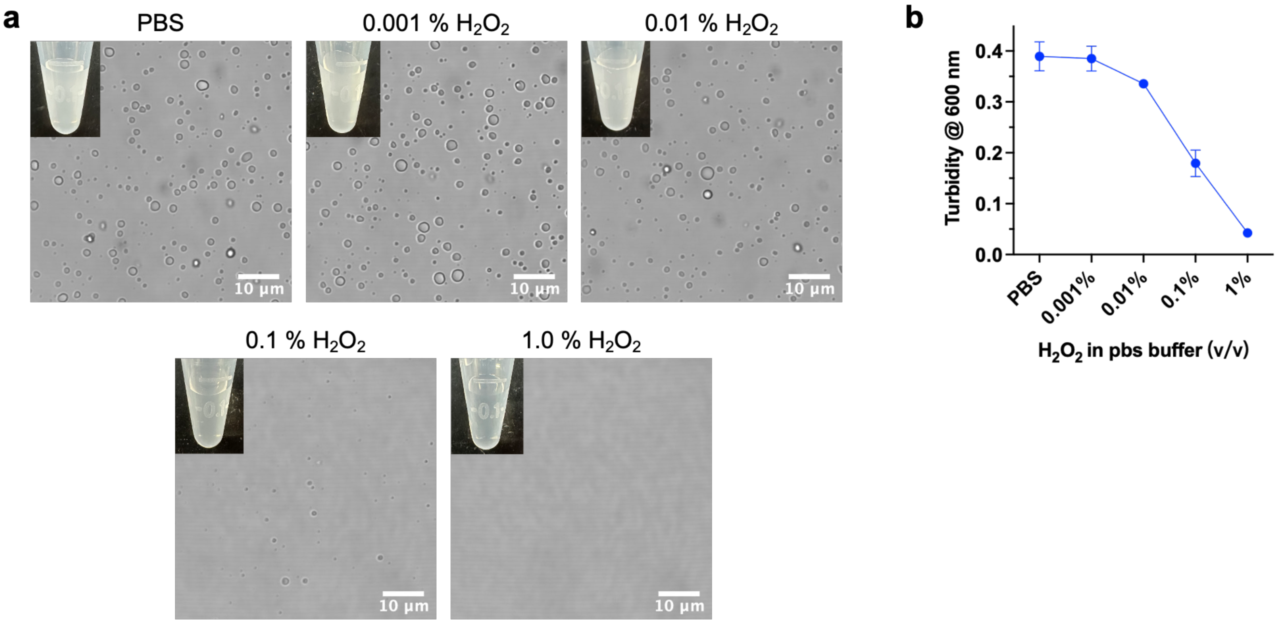

Figure S8. Treating SR-Coa with different concentrations of hydrogen peroxides. a, Confocal images. **b,** Turbidity assay (SR-Coa: 0.25 mg/mL).

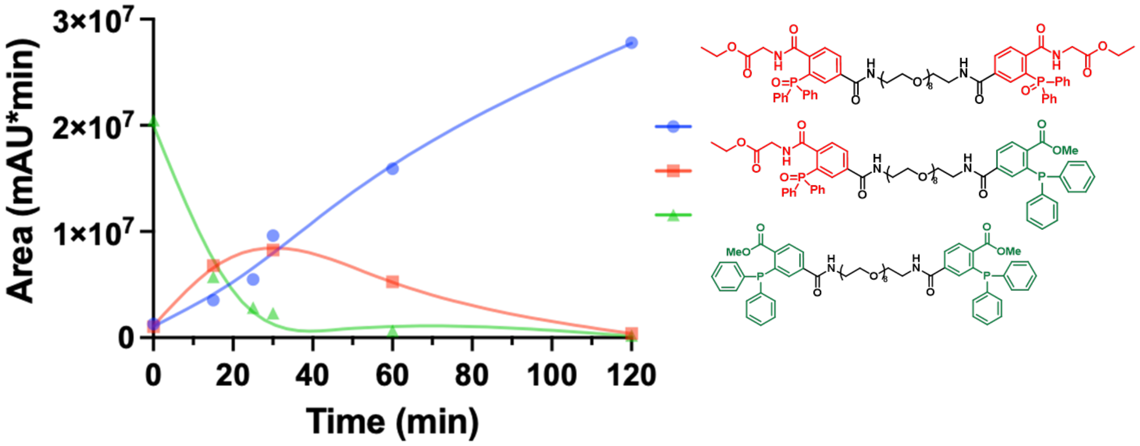

Figure S9. Quantification of the products during the reaction.

A)

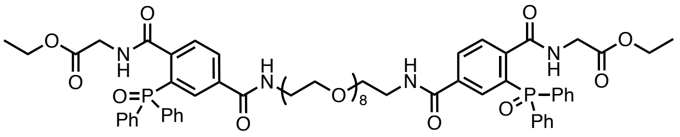

6e

B)

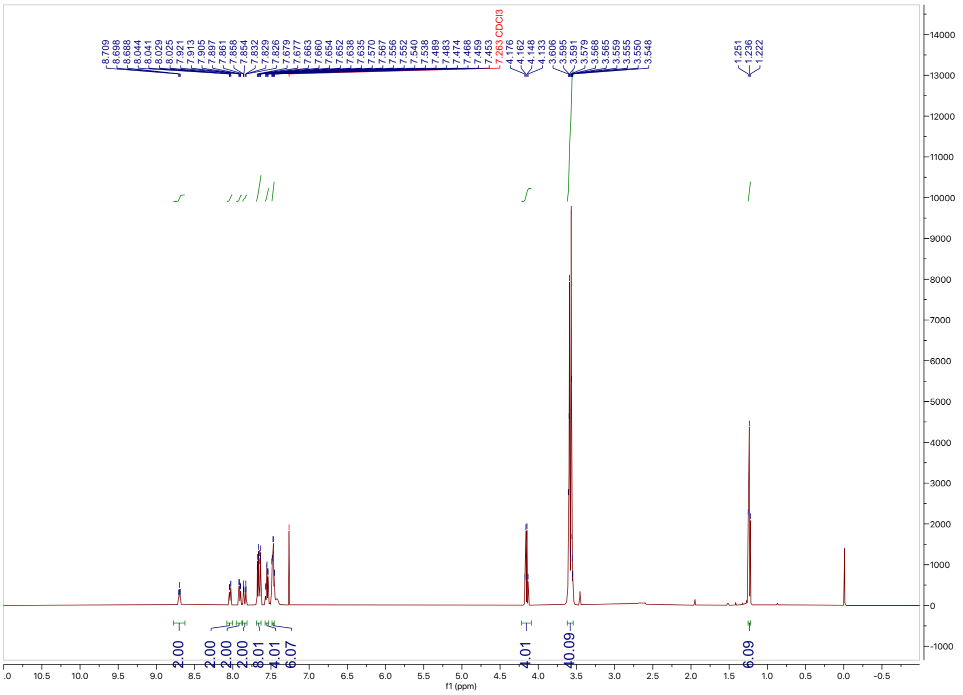

C)

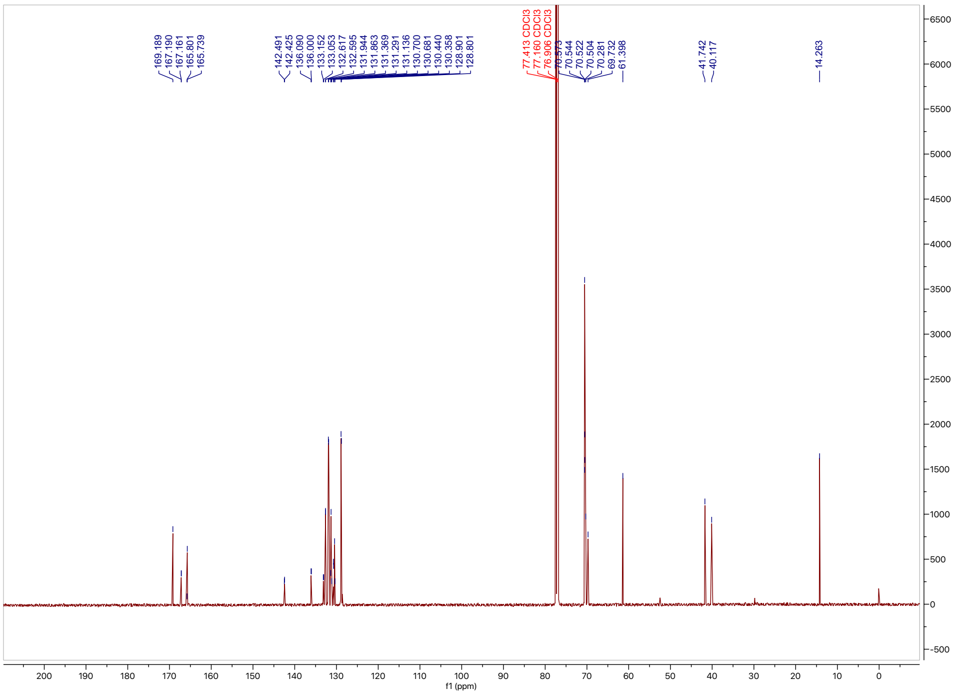

Figure S10. NMR spectra of 6e. A) Structure of **6e**. **B)** ^{1}H-NMR (500 MHz, $CDCl_3$) spectrum of **6e**. **C)** ^{13}C NMR (126 MHz, $CDCl_3$) spectrum of **6e**.

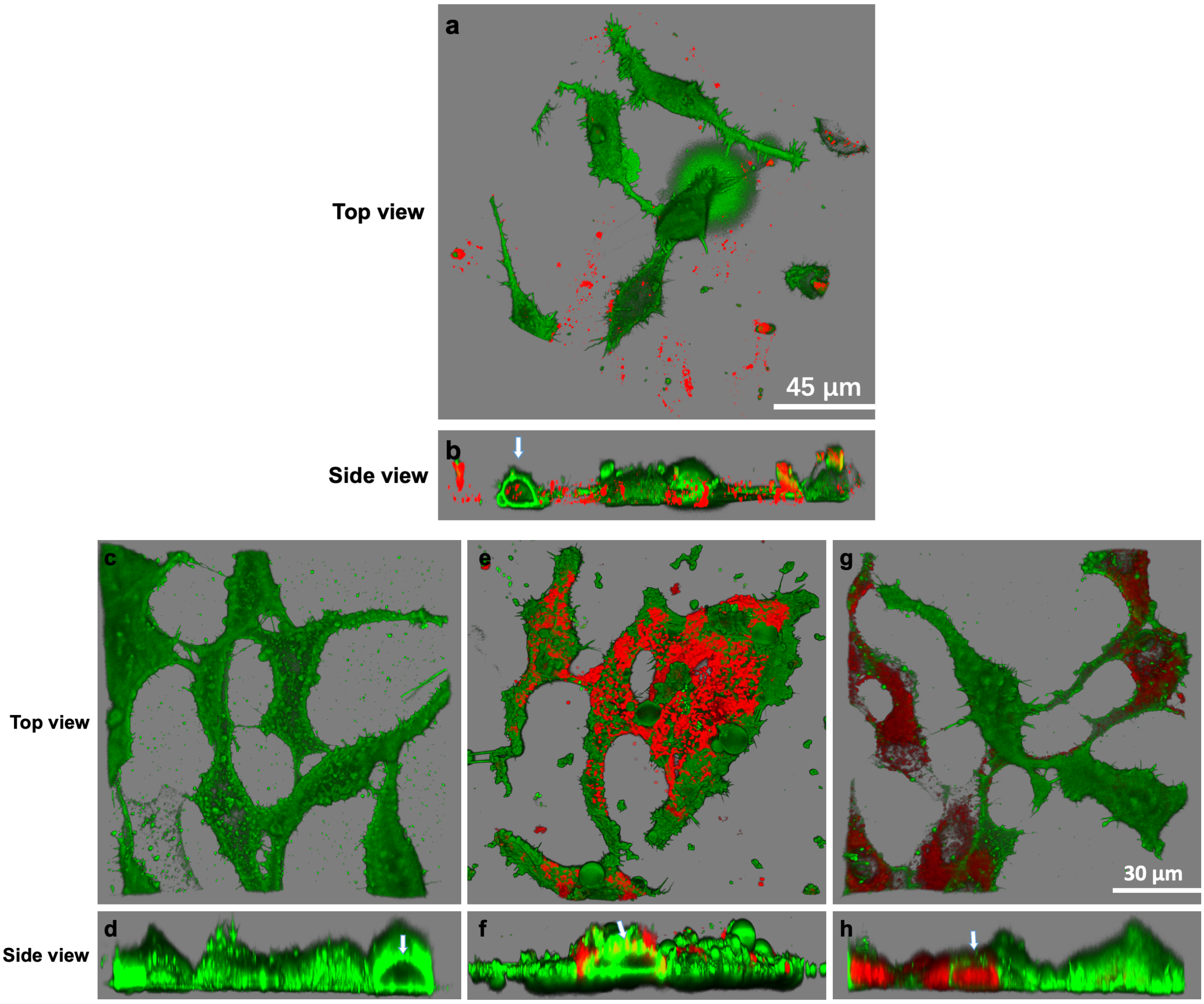

Figure S11. 3D confocal microscopic images of HeLa cells treated with SR-Coa coacervates. Top **(a)** and side **(b)** view images of cells with cell membranes co-stained with DiO dye. Green: DiO, red: Nile red-containing coacervates. 3D confocal images of HeLa cells treated with PBS **(c, d)** and 1.5 µM Nile red (without **SR-Coa**) for 4 h **(e, f)** and 24 h **(g, h)**. Scale bar: **a-b**: 45 µm, **c-h**: 30 µm. The fluorescence of DiO and Nile red was detected using the excitation/emission wavelengths of 480 nm / 510 nm and 549 nm / 628 nm, respectively.

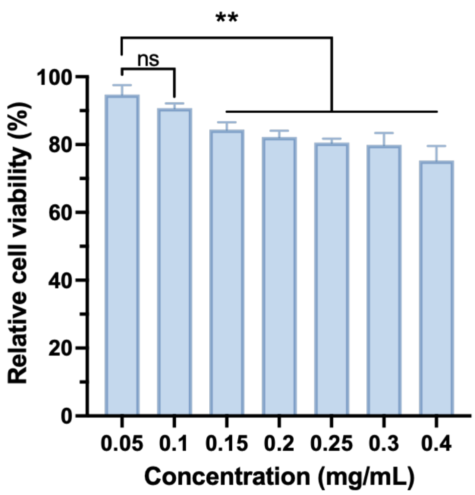

Figure S12. Cytotoxicity of SR-Coa by the CCK8 assay. Relatively low cell cytotoxicity between 0.05 to 0.3 mg/mL was observed (relative cell viability ⩾ 80%). Statistical significance was calculated using the t-test. ns: no significant difference between data, $^{**}P < 0.01$. Data are presented as the mean ± s.d. of n = 3 independent experiments.

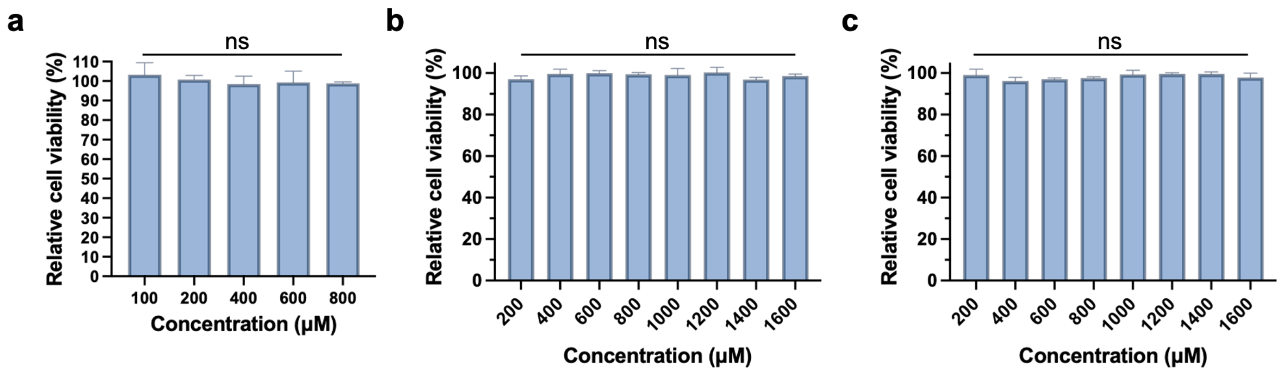

Figure S13. Cytotoxicity of the ethyl azidoacetate by the CCK8 assay. a, Ethyl azidoacetate alone incubated with the HeLa cell with different concentrations for 24h. **b,** After **SR-Coa** entered the HeLa cell, ethyl azidoacetate with different concentrations was added, and incubated for another 24h. **c**, After **SR-Coa** coacervates entered the HeLa cell, ethyl azidoacetate with different concentrations was added, and incubated for another 48h. Statistical significance was calculated using the t-test. ns: no significant difference between data. Data are presented as the mean ± s.d. of n > 3 independent experiments.

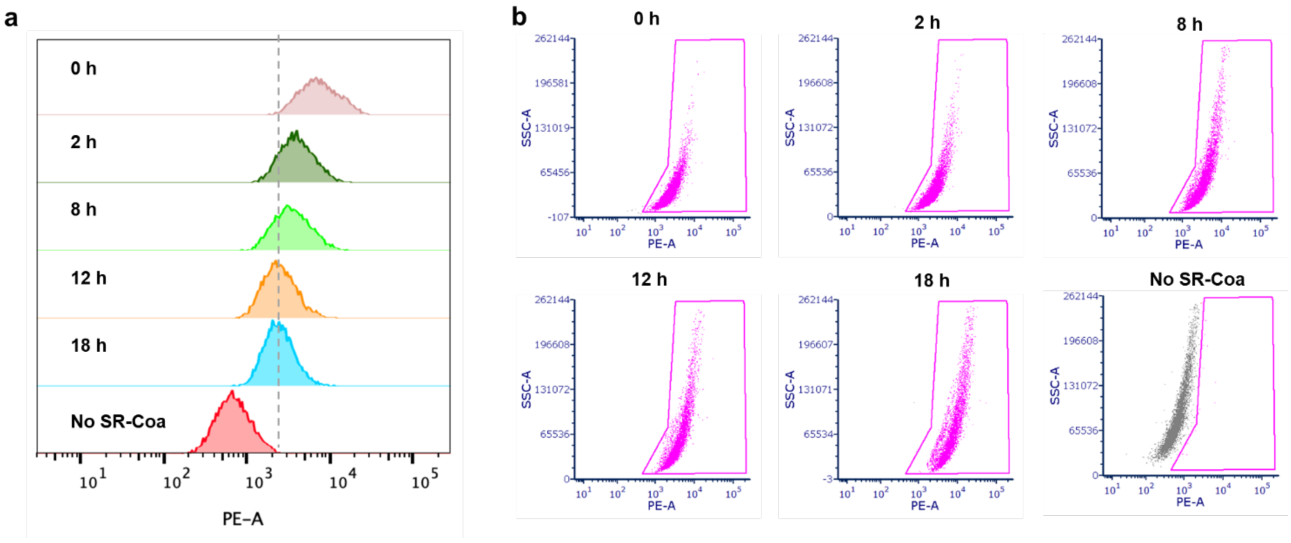

Figure S14. Fluorescence-activated cell sorting (FACS) study of azidoacetate responsiveness of SR-Coa-treated cells. a, Fluorescence intensity change in HeLa cells. **b,** Percentage of Nile red-stained **SR-Coa** positive HeLa cells. HeLa cells were treated with Nile red-stained **SR-Coa** followed by the addition of ethyl azidoacetate (450 µM) for 2 h, 8 h, 12 h, and 18 h.

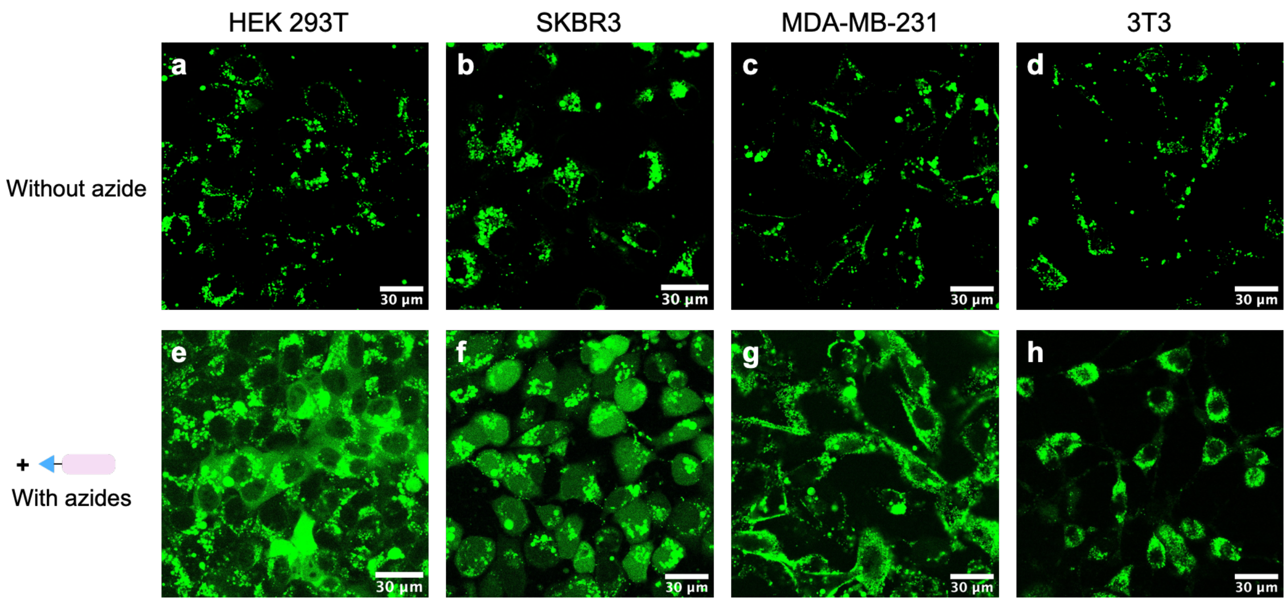

Figure S15. Intracellular delivery of AF488-BSA to different cell lines. Confocal microscopic images of HEK 293T **(a, e)**, SKBR3 **(b, f)**, MDA-MB-231 **(c, g)**, and 3T3 **(d, h)** cell lines treated with AF488-BSA-loaded coacervates (green) without azide **(a-d)** and with azide **(e-h)** (AF488: Alexa Fluor 488).

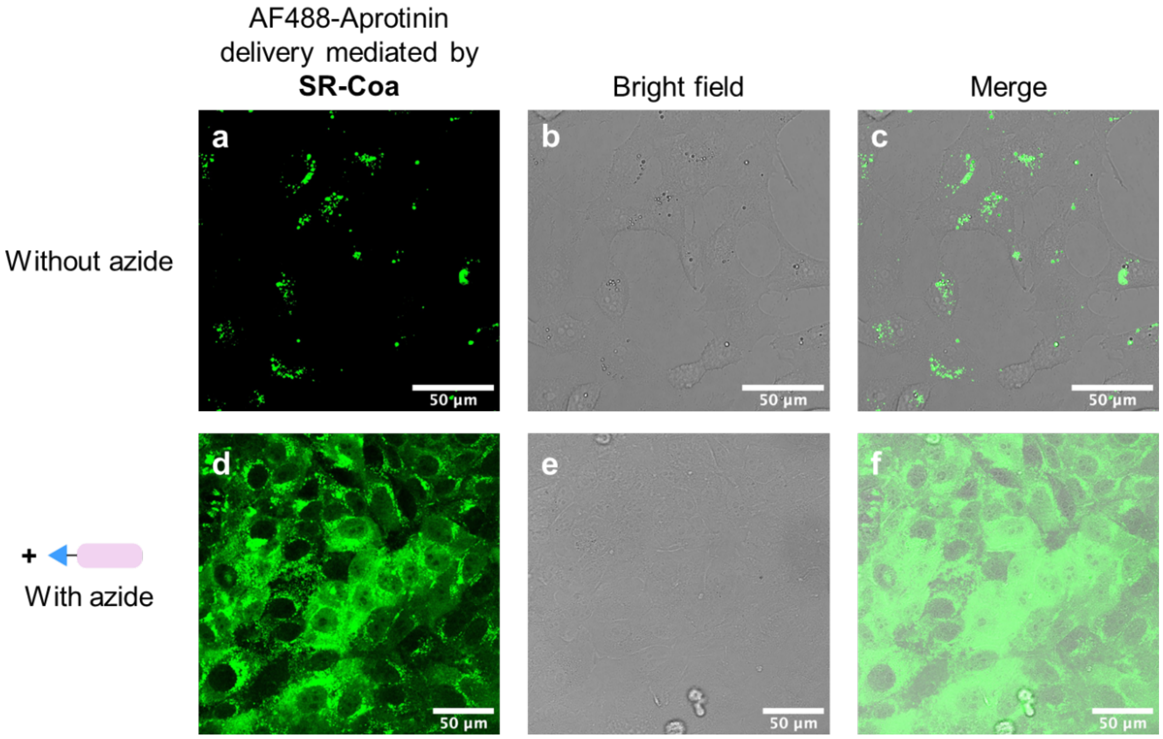

Figure S16. Intracellular delivery of AF488-aprotinin. Confocal microscopic images of HeLa cells treated with AF488-Aprotinin-loaded coacervates (green) without azide **(a-c)** and with azide **(d-f)**.

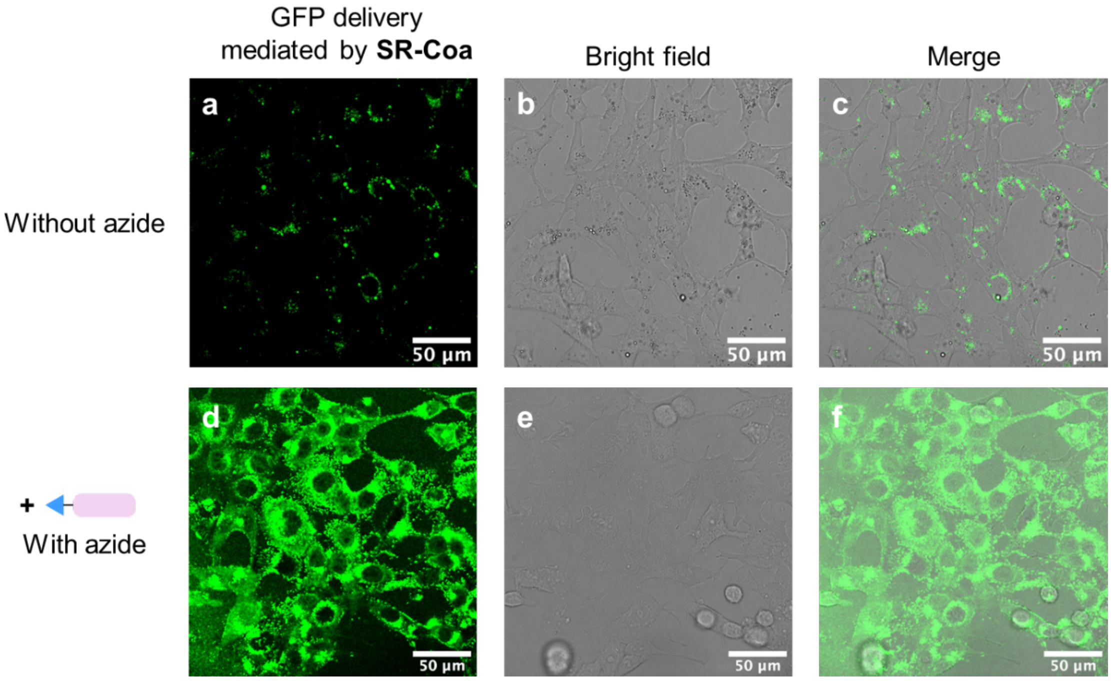

Figure S17. Intracellular delivery of GFP. Confocal microscopic images of HeLa cells treated with GFP-loaded coacervates (green) without azide **(a-c)** and with azide **(d-f)**.

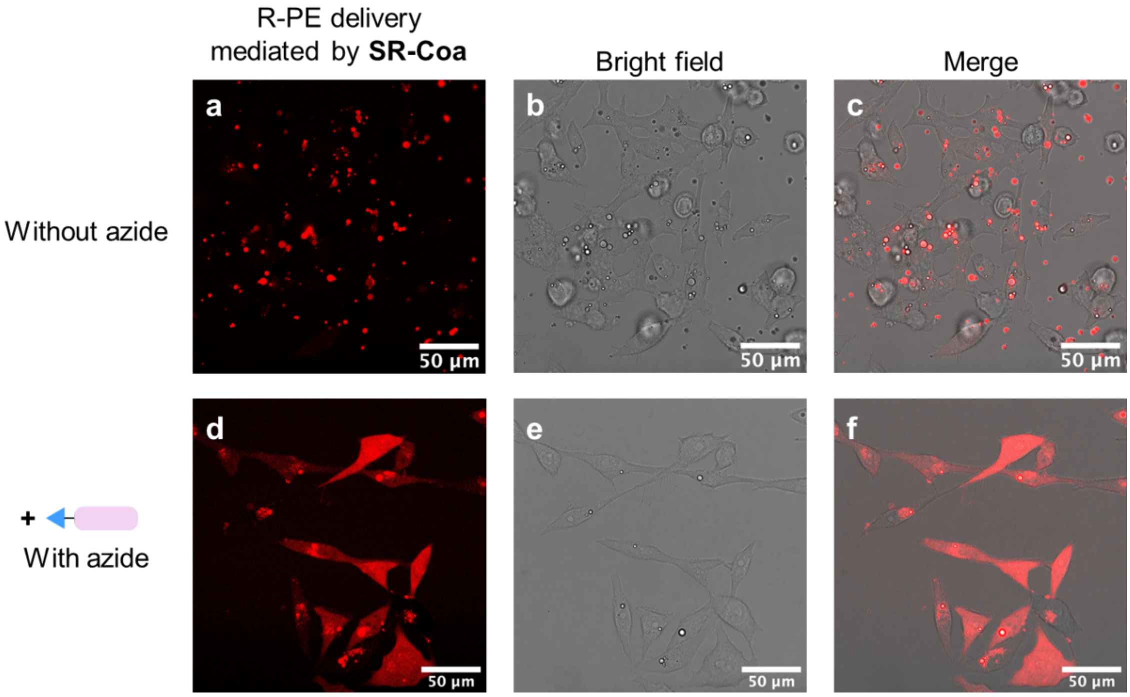

Figure S18. Intracellular delivery of R-PE. Confocal microscopic images of HeLa cells treated with R-PE-loaded coacervates (green) without azides **(a-c)** and disassembling by azides **(d-f)**.

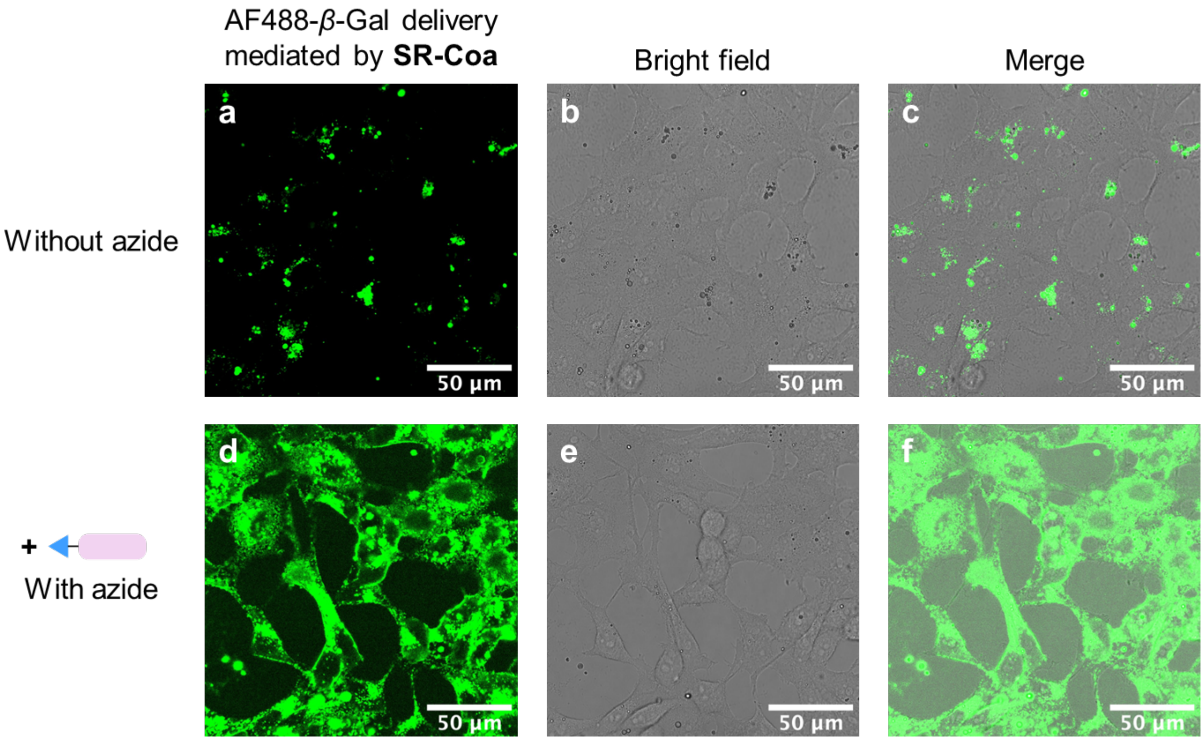

Figure S19. Intracellular delivery of AF488-β-Gal. Confocal microscopic images of HeLa cells treated with AF488-β-Gal-loaded coacervates (green) without azides **(a-c)** and disassembling by azides **(d-f)**.

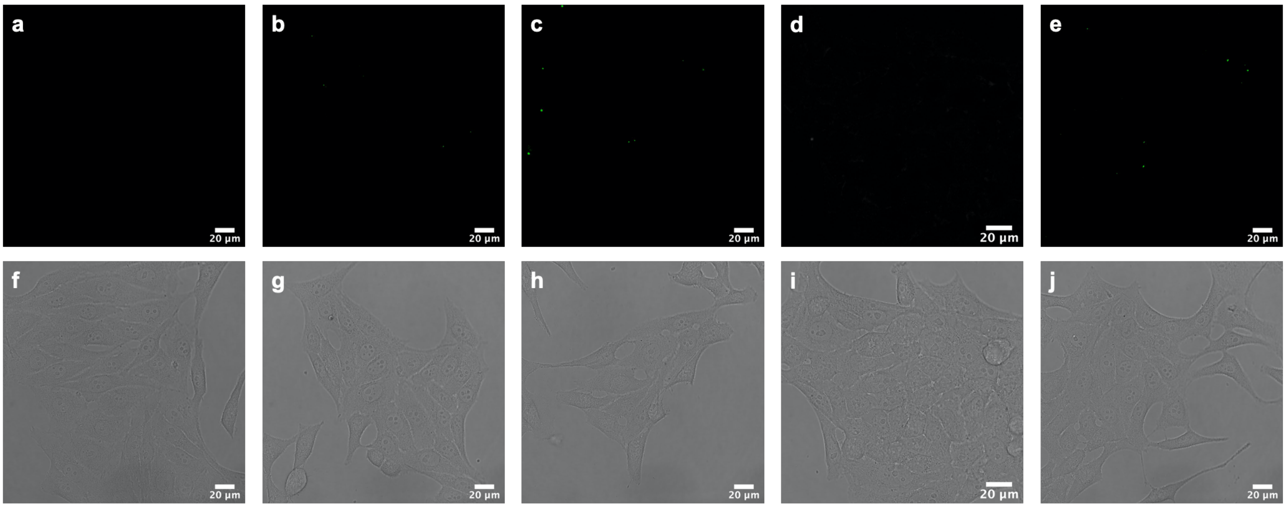

Figure S20. Fluorescent proteins alone did not enter cells without SR-Coa coacervates. Fluorescent (**a-e**) and brightfield (**f-j**) images of HeLa cells treated with free AF488-BSA, AF488-Aprotinin, GFP, R-PE, and AF488-β-Gal without **SR-Coa** coacervates (AF488 = Alexa Fluor™ 488).

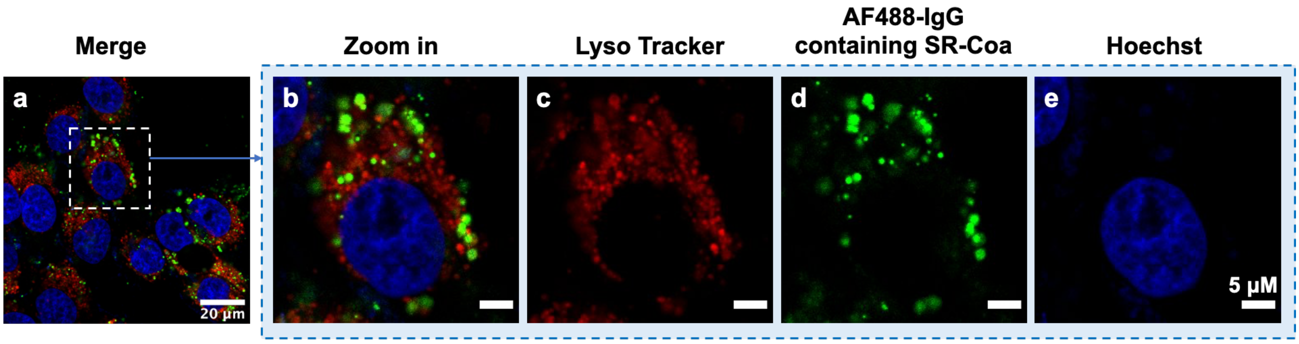

Figure S21. Confocal fluorescent microscopy images for cells incubated with AF488-IgG/SR-Coa and LysoTrackers. The fluorescence of LysoTracker, AF488-IgG, and Hoechst 34580 was detected using excitation/emission wavelengths of 577 nm / 590 nm, 488 nm / 519 nm, and 392 nm / 440 nm, respectively.

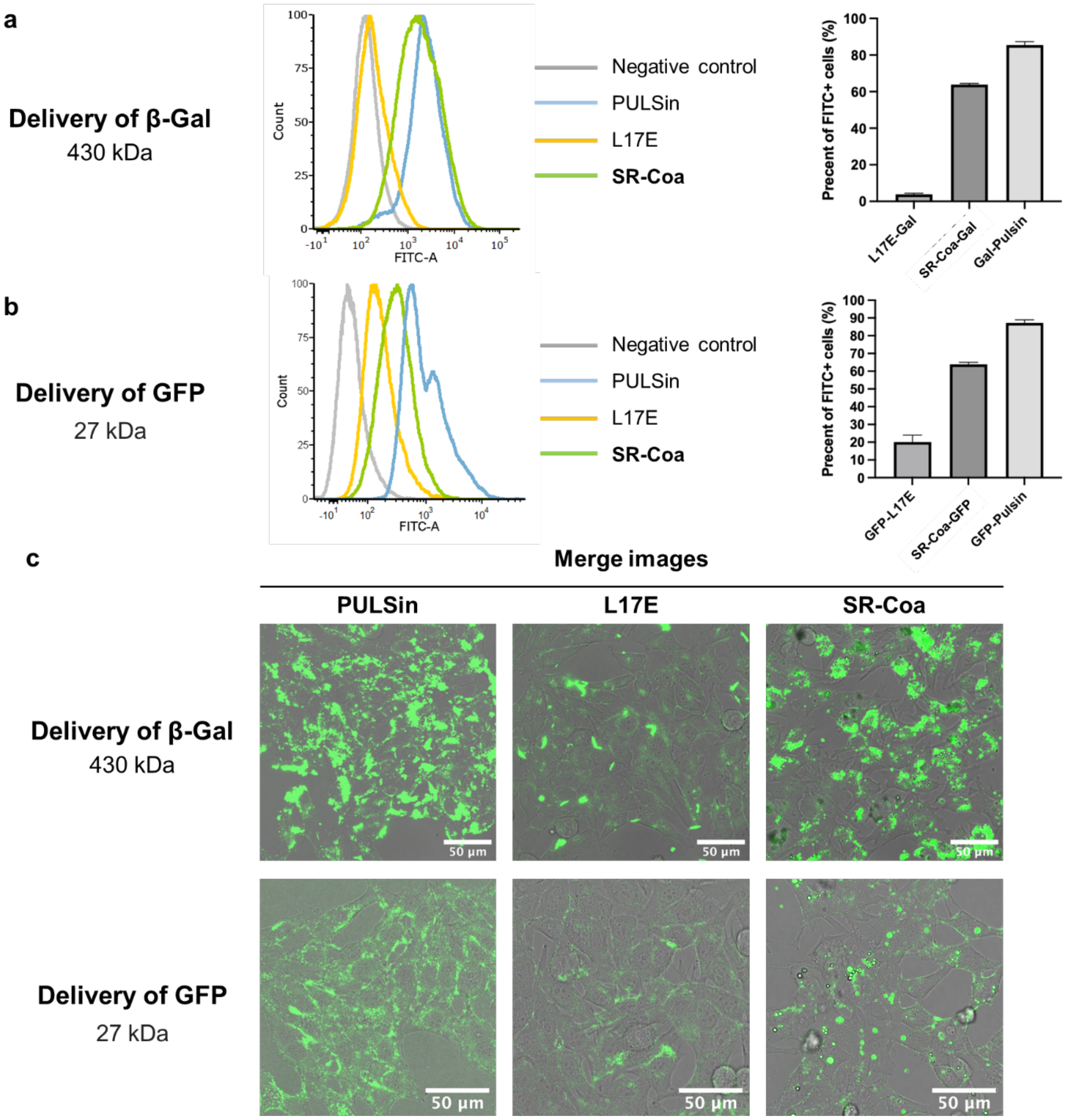

Figure S22. Comparison of the efficiency of protein delivery by different vehicles into HeLa cells. **a,** Fluorescence-activated cell sorting (FACS) analyses of AF488-β-Gal delivery. **b,** FACS analysis of GFP delivery. **c**, Confocal images of the AF488-β-Gal delivery mediated by **SR-Coa** coacervates, L17E (cell-penetrating peptides), and PULSin (commercial reagents used for protein delivery), respectively.

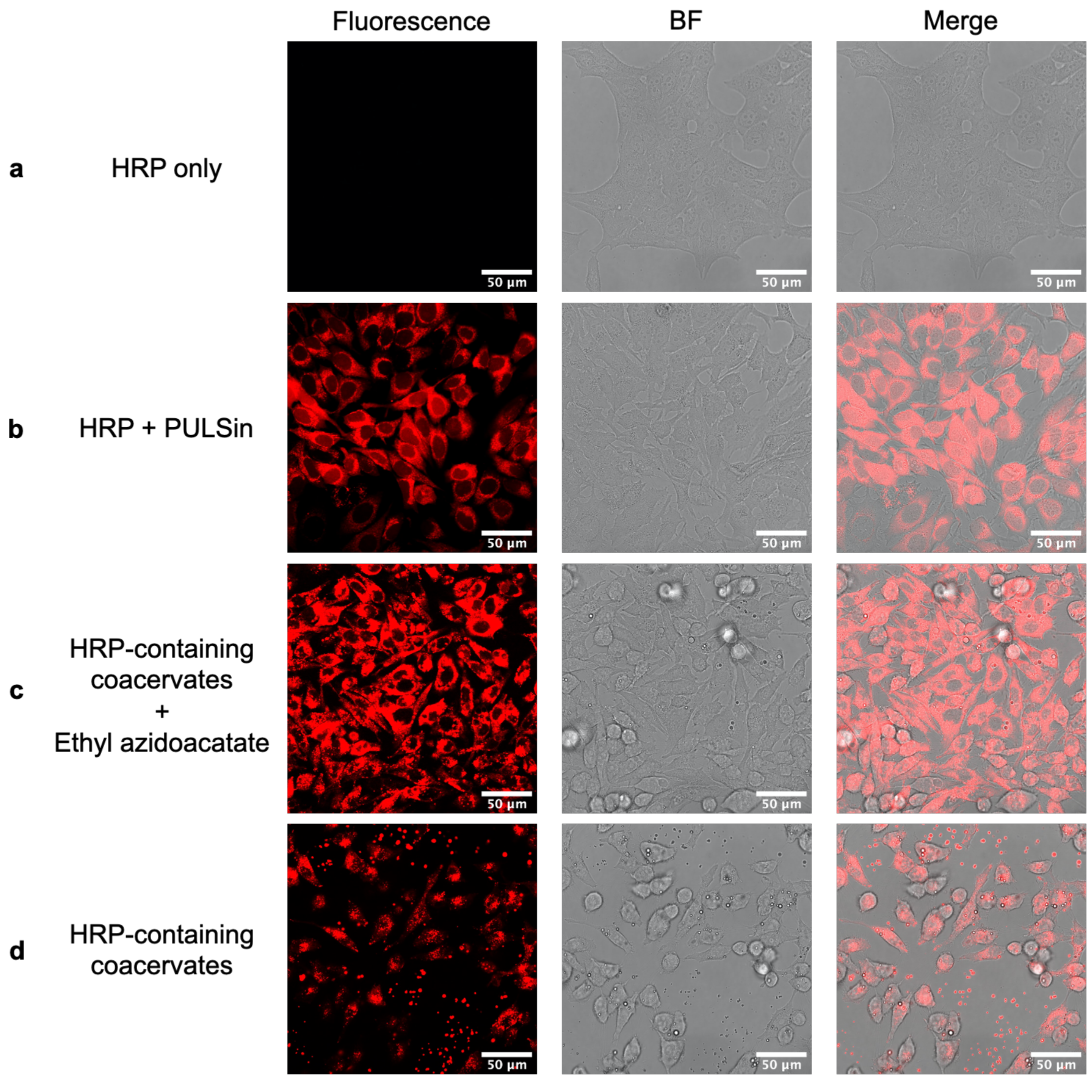

Figure S23. Intracellular HRP enzymatic activity. a-d, All groups were incubated in PBS containing Amplex Red (50 μM) and hydrogen peroxide (500 μM) for 30 min before observing under the microscopy. **a**, Confocal microscopy images of HeLa cells treated with HRP only. **b**, Commercial reagents PULSin was used as positive controls. **c**, Confocal microscopy images of HeLa cells treated with HRP-containing coacervates for 24 h, and then addition of the ethyl azidoacatate incubated for another 12 h. **d**, Confocal microscopy images of HeLa cells treated with HRP-containing coacervates for 24 h. The fluorescence of Resorufin was detected using the excitation/emission wavelengths of 587 nm / 610 nm.

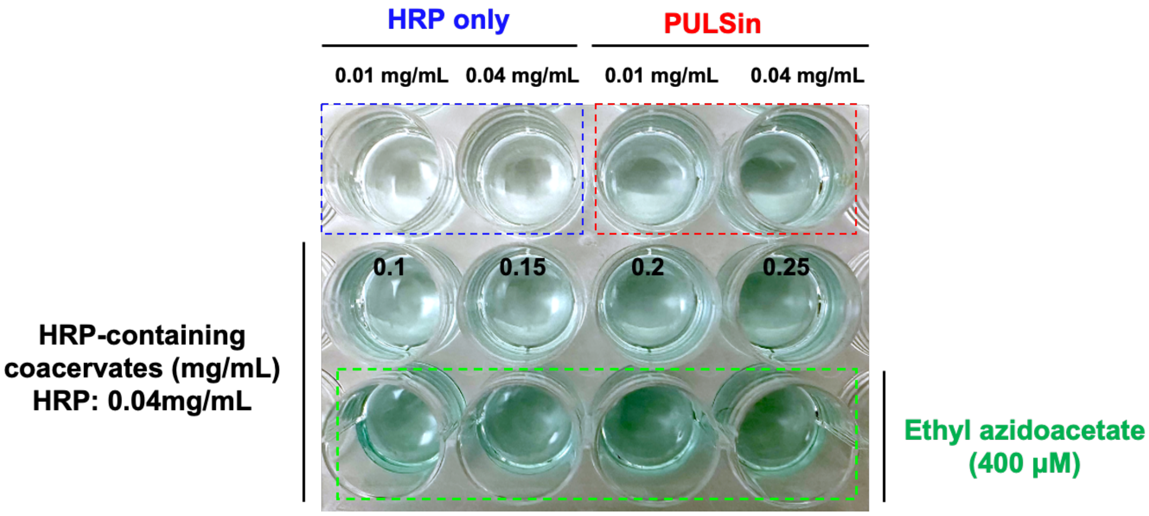

Figure S24. Intracellular HRP enzymatic activity analysis by TMB assay. Control groups: only add HRP enzymes (0.01 mg/mL and 0.04 mg/mL). Positive groups: delivery of HRP enzymes by commercial reagents PULSin (0.01 mg/mL and 0.04 mg/mL). Experimental groups: HRP enzymes (0.04 mg/mL) were delivered by **SR-Coa** coacervates with different concentrations. After adding azides, the solution displayed a darker blue color. TMB: 3,3'5,5′-Tetramethylbenzidine.

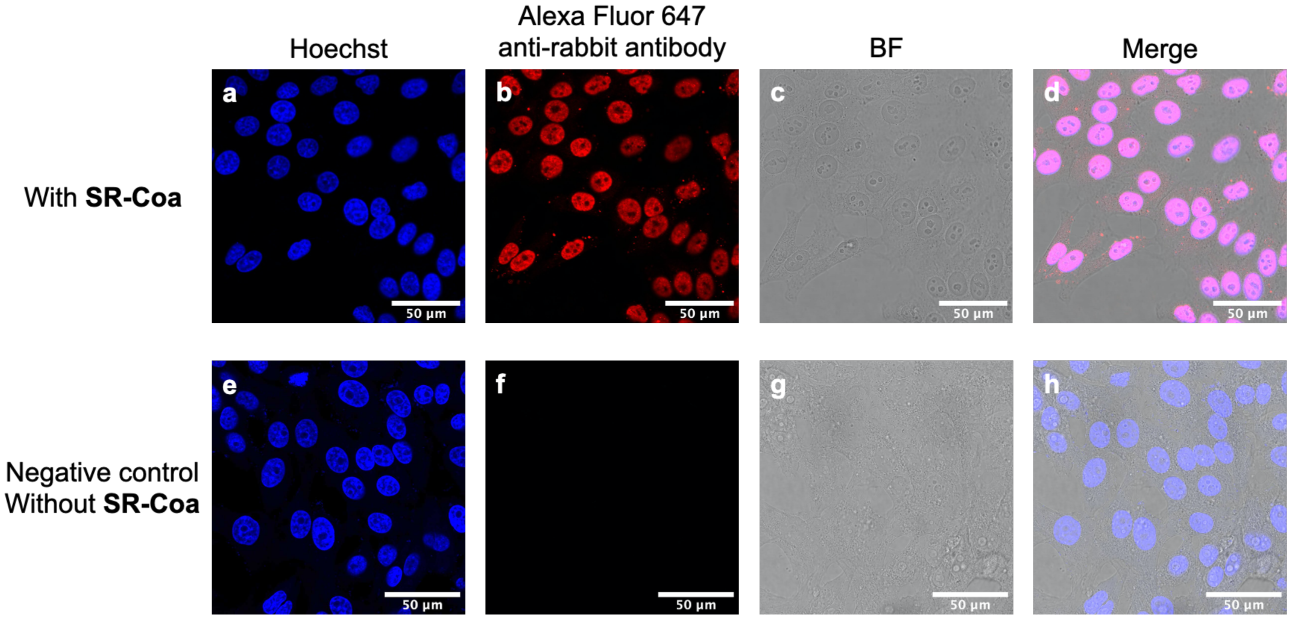

Figure S25. Nucleoplasm localization of a coacervate-delivered anti-EZH2 antibody without permeabilization. Confocal microscopic images of HeLa cells treated the second antibody, Alexa-647 anti-rabbit antibody (red) and Hoechst coacervates (blue). The first antibody was delivered into the HeLa cells by **SR-Coa** coacervates (**a-d**) and without **SR-Coa** coacervates (**e-h**). The fluorescence of Alexa Fluor 647 anti-rabbit antibody and Hoechst 34580 was detected using the excitation/emission wavelengths of 647 nm / 680 nm and 392 nm / 440 nm, respectively.

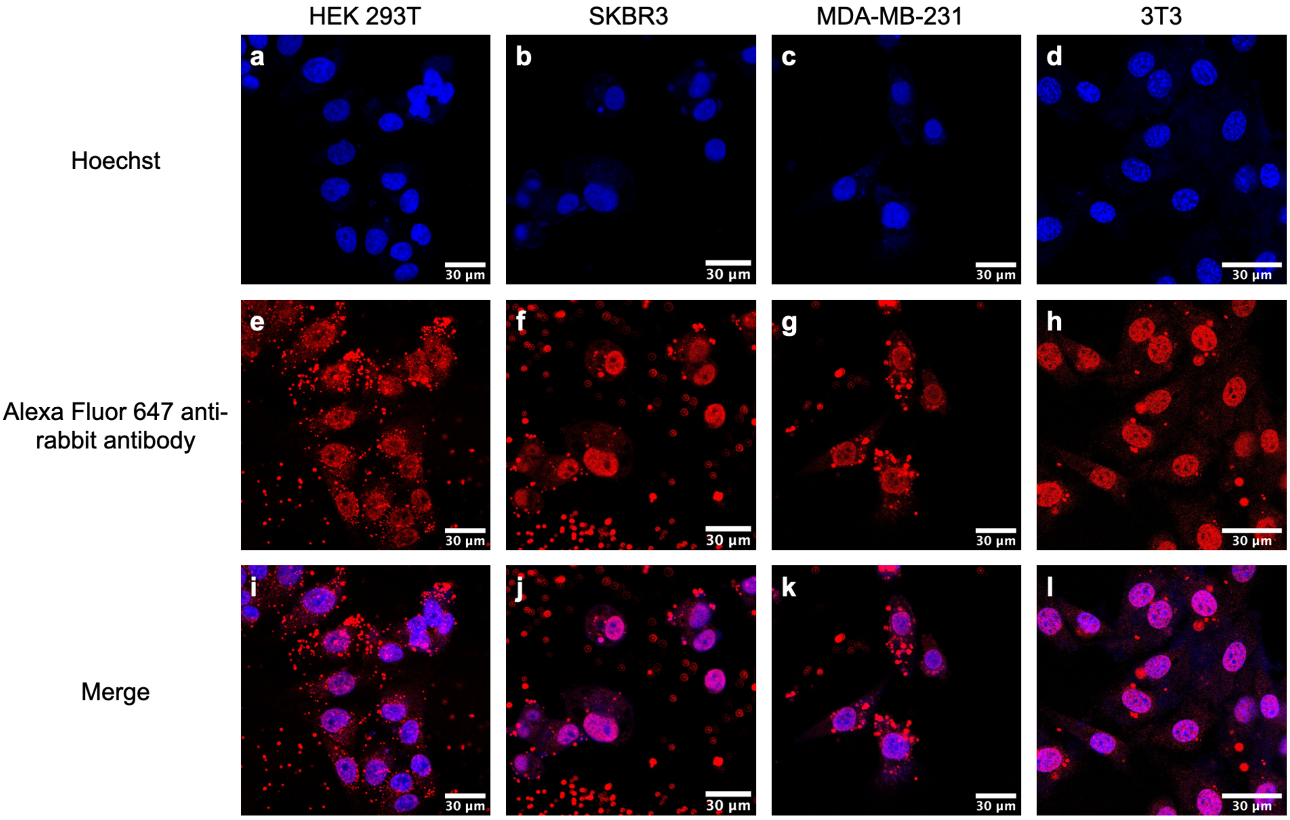

Figure S26. Delivery of anti-EZH2 antibody into HEK 293T, SKBR3, MDA-MB-231, and 3T3 cell lines by SR-Coa. Confocal microscopic images of HEK 293T (**a, e, i**), SKBR3 (**b, f, j**), MDA-MB-231 (**c, g, k**), and 3T3 (**d, h, l**) cell lines treated with the second antibody, Alexa Fluor 647 anti-rabbit antibody (red) and Hoechst coacervates (blue). The first antibody was delivered into these cells by **SR-Coa** coacervates. The fluorescence of Alexa Fluor 647 anti-rabbit antibody and Hoechst 34580 was detected using the excitation/emission wavelengths of 647 nm / 680 nm and 392 nm / 440 nm, respectively.

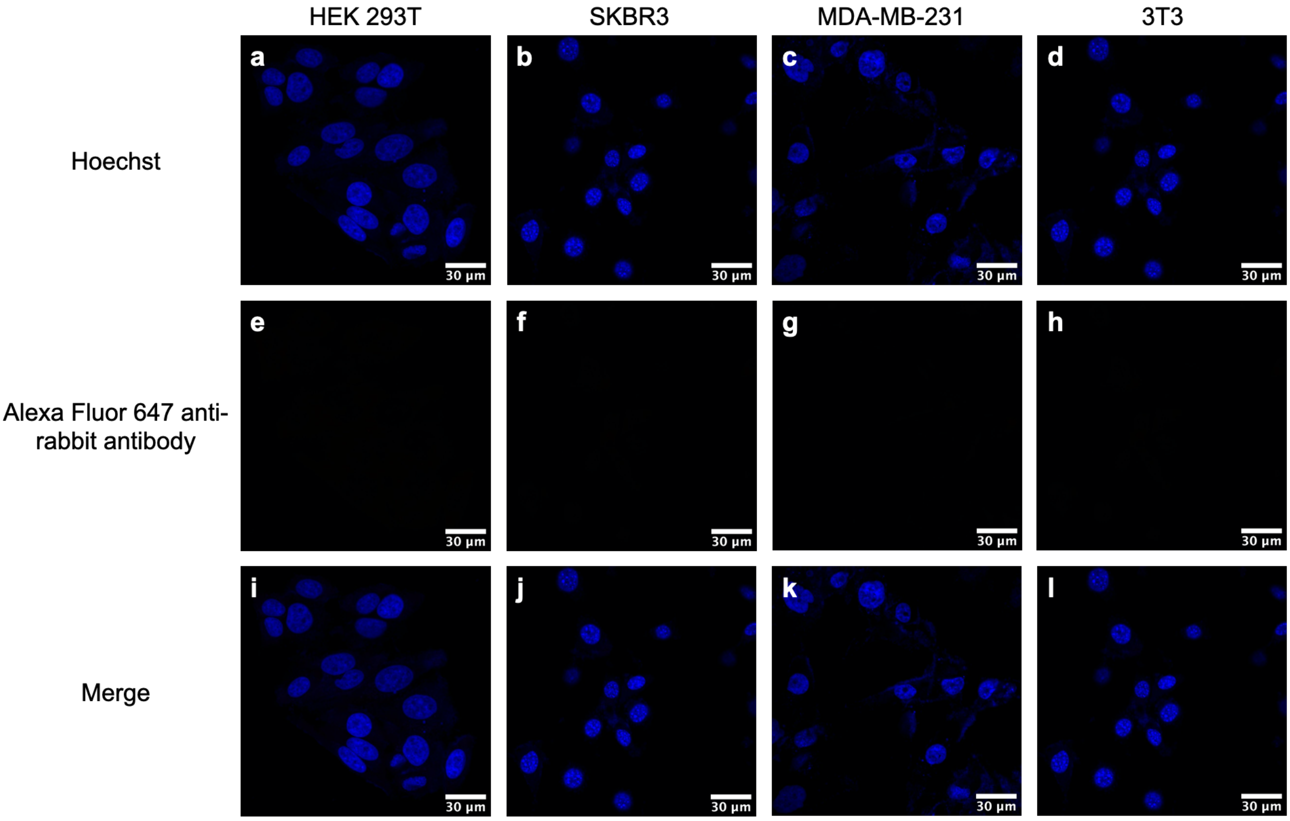

Figure S27. Anti-EHZ2 antibody alone did not enter 293T, SK-BR-3, MDA-MB-231, and 3T3 cell lines without SR-Coa. Confocal microscopic images of 293T (**a, e, i**), SKBR3 (**b, f, j**), MDA-MB-231 (**c, g, k**), and 3T3 (**d, h, l**) cell lines treated with the second antibody, Alexa Fluor 647 anti-rabbit antibody (red) and Hoechst coacervates (blue). The fluorescence of Alexa Fluor 647 anti-rabbit antibody and Hoechst 34580 was detected using the excitation/emission wavelengths of 647 nm / 680 nm and 392 nm / 440 nm, respectively.

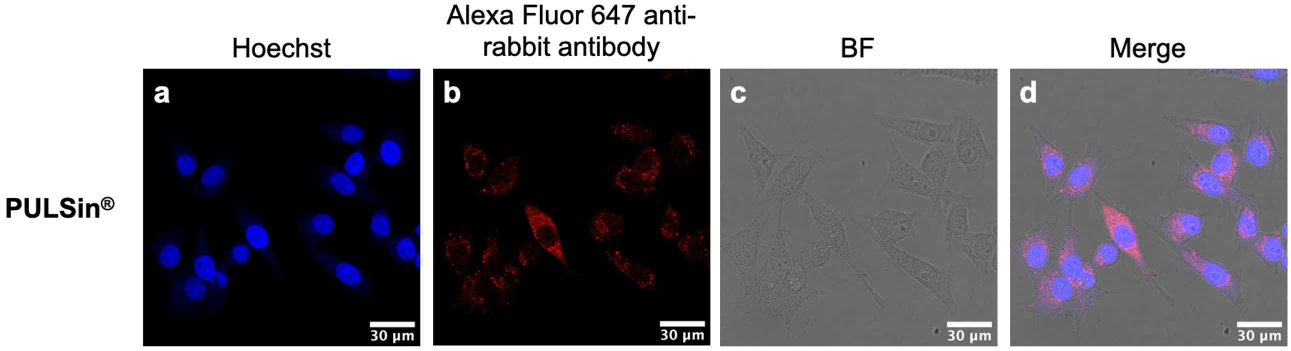

Figure S28. Delivery of anti-EZH2 antibody into HEK 293T by PULsin®. Confocal microscopic images of 293T cells treated the second antibody, Alexa-647 anti-rabbit antibody (red) and Hoechst coacervates (blue). The first antibody was delivered into the 293T cells by PULsin® (**a-d**). The fluorescence of Alexa Fluor 647 anti-rabbit antibody and Hoechst 34580 was detected using the excitation/emission wavelengths of 647 nm / 680 nm and 392 nm / 440 nm, respectively.

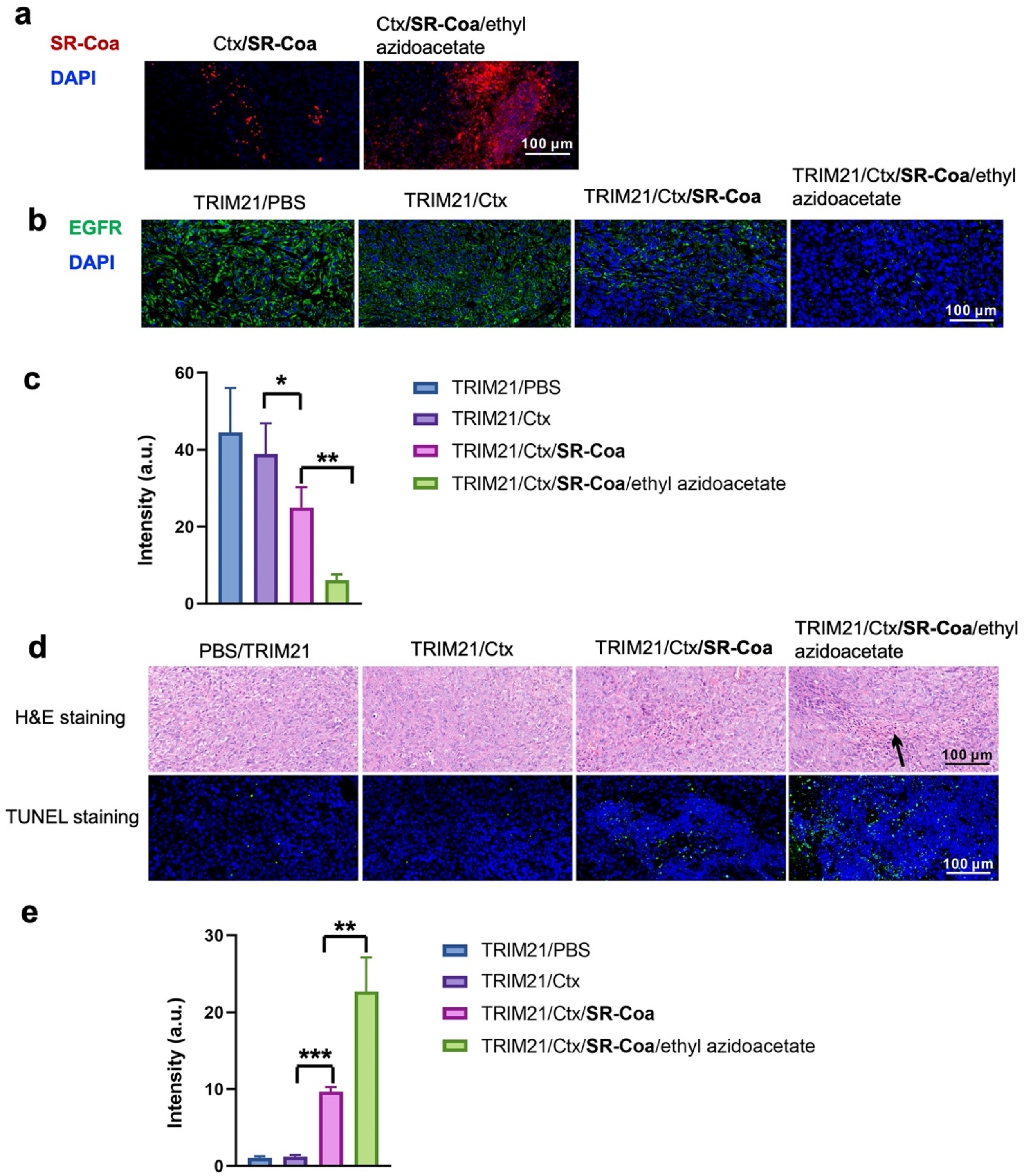

Figure S29. Coacervate-mediated EGFR degradation in *vivo*. a. Fluorescent images of tumor tissues injected with Nile red-stained **SR-Coa** treated with or without ethyl azidoacetate. **b.** EGFR expression levels of tumor tissue sections in different treatment groups based on immunofluorescent staining. **c**. Quantification of the EGFR expression levels based on the fluorescence of EGRF antibody

in selected representative images. **d.** Images of tumor tissue sections stained with hematoxylin/eosin (H&E) and TdT-mediated dUTP Nick-End Labeling (TUNEL), respectively. The arrow points to signs of apoptosis. **e**. Quantification of the fluorescent signals in selected representative TUNEL staining images. Nile red, FITC, and DAPI were detected using the excitation/emission wavelengths of 549 nm / 628 nm, 488 nm / 520 nm, and 364 nm / 454 nm, respectively. * : $p<0.05$; ** : $p<0.01$; *** : $p<0.001$.

Characterization Data

Dimethyl 4,4'-(5,8-dioxa-2,11-diazadodecanedioyl)bis(2-(diphenylphosphaneyl)benzoate) (**6a**)

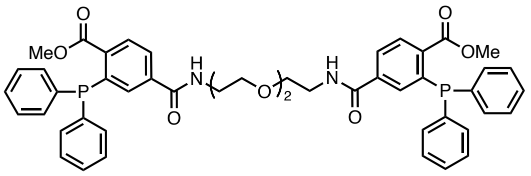

6a

^{1}H NMR (500 MHz, $CDCl_3$) δ 8.06 (dd, *J* = 8.1, 3.6 Hz, 2H), 7.75 (dd, *J* = 8.1, 1.8 Hz, 2H), 7.35 – 7.32 (m, 12H), 7.31 – 7.26 (m, 8H), 6.41 (t, *J* = 5.5 Hz, 2H), 3.74 (s, 6H), 3.56 – 3.47 (m, 12H). **^{13}C NMR** (126 MHz, $CDCl_3$) δ 166.8, 166.8, 166.6, 141.8, 141.6, 137.3, 137.3, 136.9, 136.8, 134.1, 133.9, 132.8, 131.0, 131.0, 129.1, 128.8, 128.7, 126.8, 77.4, 77.2, 76.9, 70.3, 69.8, 52.4, 51.0, 39.9. **HRMS (ESI)** m/z: $[M+H]^+$ Calcd. for 841.2802; Found 841.2808.

Dimethyl 4,4'-(5,8,11,14-tetraoxa-2,17-diazaoctadecanedioyl)bis(2-(diphenylphosphaneyl)benzoate) (**6b**)

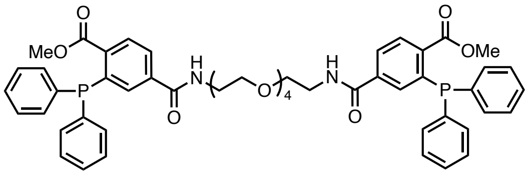

6b

^{1}H NMR (500 MHz, $CDCl_3$) δ 8.04 (dd, *J* = 8.0, 3.6 Hz, 2H), 7.77 (dd, *J* = 8.1, 1.8 Hz, 2H), 7.39 (dd, *J* = 3.8, 1.8 Hz, 2H), 7.35 – 7.30 (m, 12H), 7.30 – 7.26 (m, 8H), 6.90 (t, *J* = 5.4 Hz, 2H), 3.72 (s, 6H), 3.57 (d, *J* = 10.7 Hz, 12H), 3.52 (dd, *J* = 5.4, 4.0 Hz, 4H), 3.50 – 3.46 (m, 4H). **^{13}C NMR** (126 MHz, $CDCl_3$) δ 166.9, 166.9, 166.6, 141.6, 141.4, 137.5, 137.3, 137.2, 136.7, 136.6, 134.1, 133.9, 133.2, 130.9, 130.8, 129.1, 128.8, 128.7, 126.7, 77.4, 77.2, 76.9, 70.6, 70.2, 69.9, 52.4, 51.0, 39.9. **HRMS (ESI)** m/z: $[M+H]^+$ Calcd. for 929.3327; Found 929.3325.

Dimethyl 4,4'-(5,8,11,14,17,20-hexaoxa-2,23-diazatetracosanedioyl)bis(2-(diphenylphosphaneyl)benzoate) (**6c**)

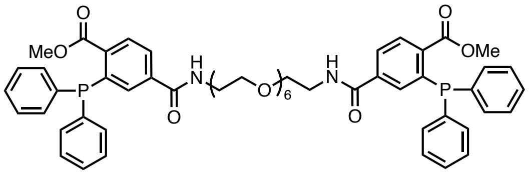

6c

^{1}H NMR (500 MHz, $CDCl_3$) δ 8.05 (dd, *J* = 8.1, 3.6 Hz, 2H), 7.80 (dd, *J* = 8.1, 1.8 Hz, 2H), 7.38 (dd, *J* = 3.8, 1.8 Hz, 2H), 7.36 – 7.30 (m, 12H), 7.30 – 7.26 (m, 8H), 6.92 (t, *J* = 5.2 Hz, 2H), 3.72 (s, 6H), 3.61 – 3.55 (m, 20H), 3.55 – 3.47 (m, 8H). ^{13}C NMR (126 MHz, $CDCl_3$) δ 166.9, 166.9, 166.6, 141.6, 141.4, 137.5, 137.3, 137.3, 136.7, 136.6, 134.1, 133.9, 133.2, 130.9, 130.9, 129.1, 128.8, 128.7, 126.8, 77.4, 77.2, 76.9, 70.6, 70.2, 69.8, 52.4, 51.0, 40.0. **HRMS (ESI)** m/z: $[M+H]^+$ Calcd. for 1017.3851; Found 1017.3853.

Dimethyl 4,4'-(5,8,11,14,17,20,23,26-octaoxa-2,29-diazatriacontanedioyl)bis(2-(diphenylphosphaneyl)benzoate) (**6d**)

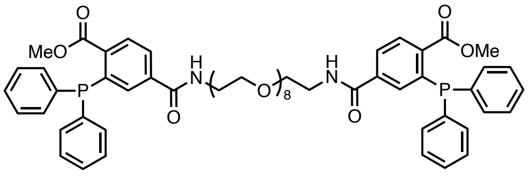

6d

^{1}H NMR (500 MHz, $CDCl_3$) δ 8.05 (dd, *J* = 8.1, 3.6 Hz, 2H), 7.81 (dd, *J* = 8.1, 1.8 Hz, 2H), 7.38 (dd, *J* = 3.8, 1.8 Hz, 2H), 7.33 (dp, *J* = 4.7, 1.6 Hz, 10H), 7.28 (dt, *J* = 7.7, 2.8 Hz, 8H), 6.95 (t, *J* = 5.1 Hz, 2H), 3.72 (s, 6H), 3.61 – 3.52 (m, 36H). **^{13}C NMR** (126 MHz, $CDCl_3$) δ 166.9, 166.9, 166.6, 141.6, 141.3, 137.5, 137.3, 137.3, 136.7, 136.6, 134.1, 133.9, 133.2, 130.9, 130.9, 129.1, 128.8, 128.7, 126.8,

77.4, 77.2, 76.9, 70.6, 70.5, 70.3, 69.8, 52.3, 40.0. **HRMS (ESI)** m/z: $[M+H]^+$ Calcd. for 1105.4375; Found 1105.4367.

Diethyl 2,2'-((4,4'-(5,8,11,14,17,20,23,26-octaoxa-2,29-diazatriacontanedioyl)bis(2-(diphenylphosphoryl)benzoyl))bis(azanediyl))diacetate (**6e**)

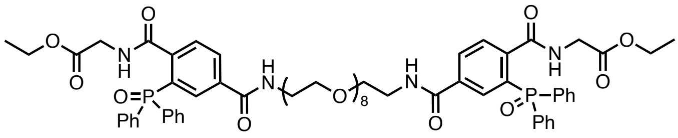

6e

^{1}H NMR (500 MHz, $CDCl_3$) δ 8.70 (t, J = 5.3 Hz, 2H), 8.03 (dd, J = 8.0, 1.6 Hz, 2H), 7.91 (dd, J = 8.0, 3.8 Hz, 2H), 7.84 (dt, J = 14.3, 1.7 Hz, 2H), 7.66 (ddd, J = 12.3, 8.3, 1.3 Hz, 8H), 7.55 (td, J = 7.4, 1.5 Hz, 4H), 7.49 – 7.46 (m, 6H), 4.15 (q, J = 7.1 Hz, 4H), 3.62 – 3.54 (m, 40H), 1.24 (t, J = 7.1 Hz, 6H). **^{13}C NMR** (126 MHz, $CDCl_3$) δ 169.2, 167.2, 167.2, 165.8, 165.7, 142.5, 142.4, 136.1, 136.0, 133.2, 133.1, 132.6, 132.6, 131.9, 131.9, 131.4, 131.3, 131.1, 130.7, 130.7, 130.4, 130.4, 128.9, 128.8, 77.4, 77.2, 76.9, 70.6, 70.5, 70.5, 70.5, 70.3, 69.7, 61.4, 41.7, 40.1, 14.3. **HRMS (ESI)** m/z: $[M+Na]^+$ Calcd. for 1301.4835; Found 1301.4814.

More characterization of the intermediates and products are shown in **Figures S30 to S33** below.

A)

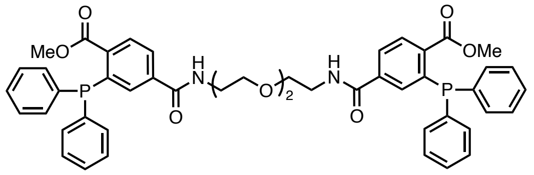

6a

B)

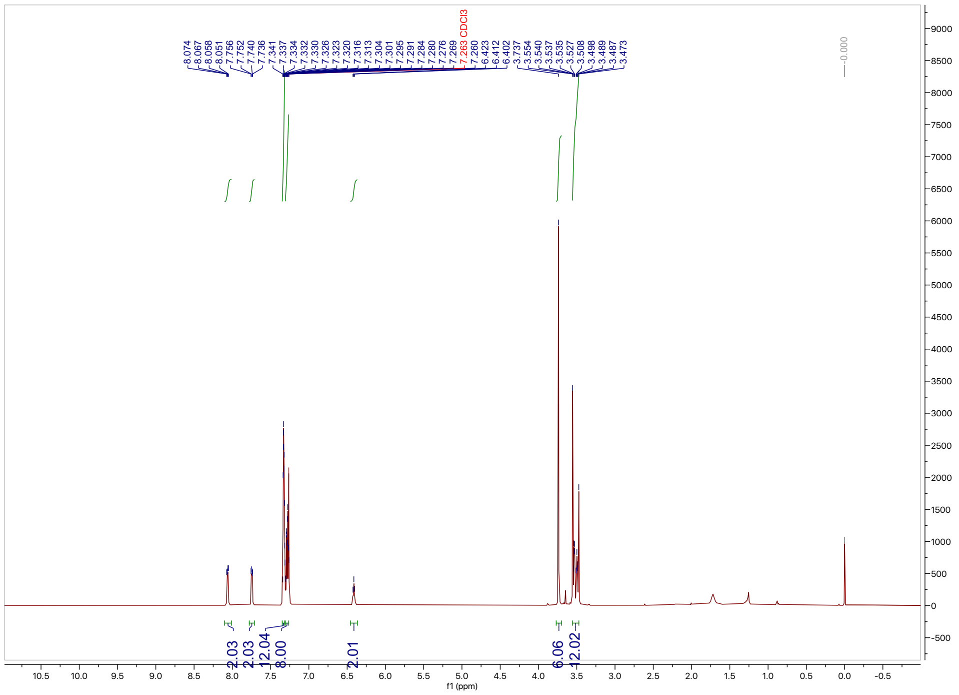

C)

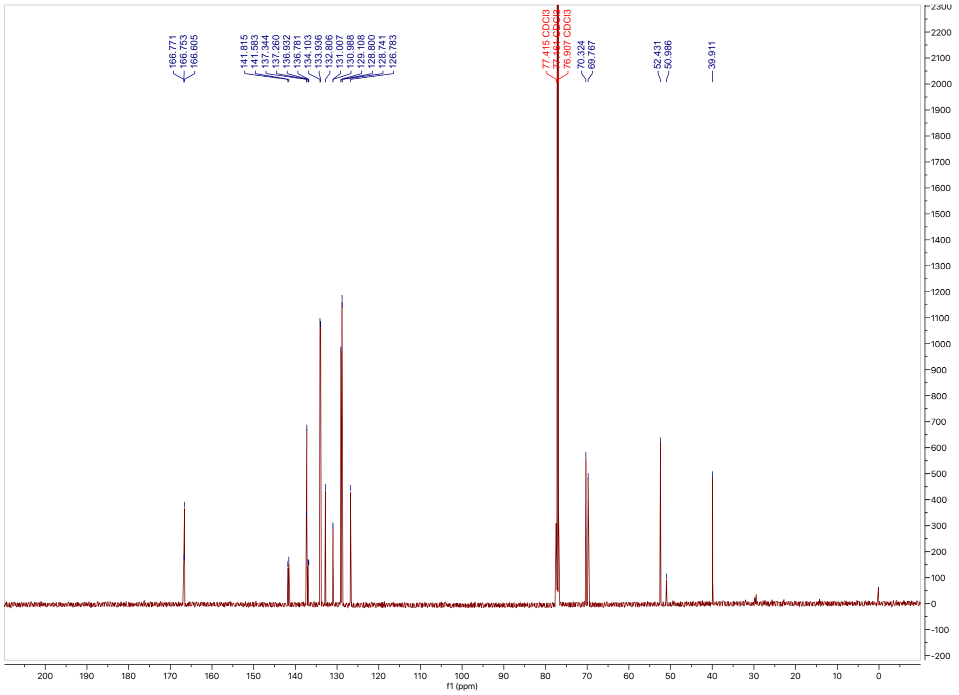

Figure S30. NMR spectra of 6a. **A)** Structure of **6a**. **B)** ^{1}H-NMR (500 MHz, $CDCl_3$) spectrum of **6a**. **C)** ^{13}C NMR (126 MHz, $CDCl_3$) spectrum of **6a**.

A)

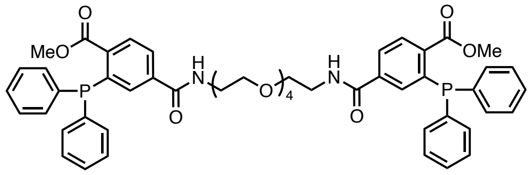

6b

B)

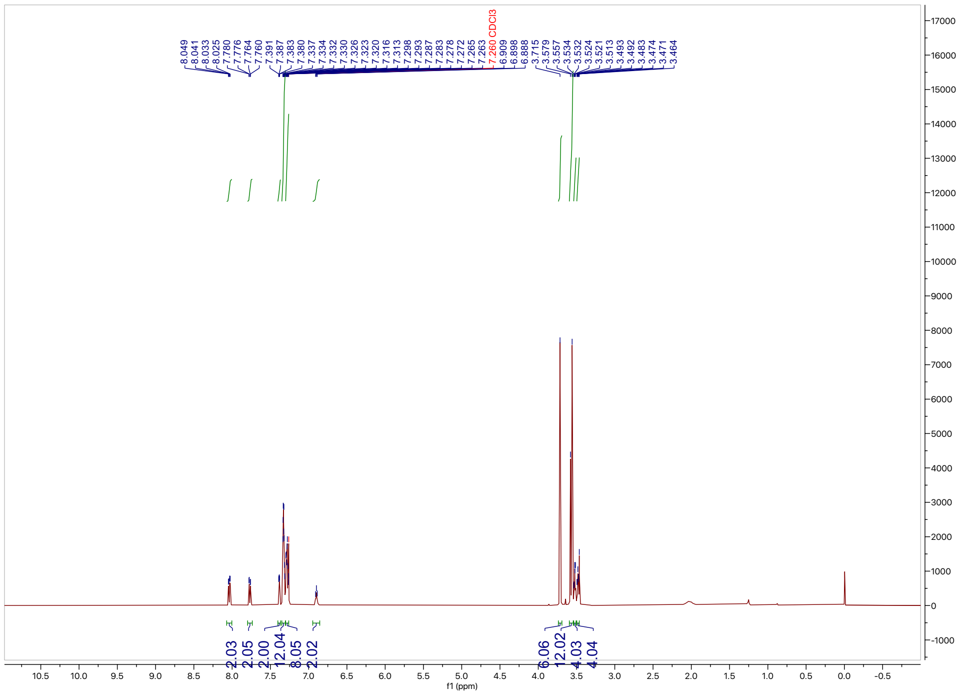

C)

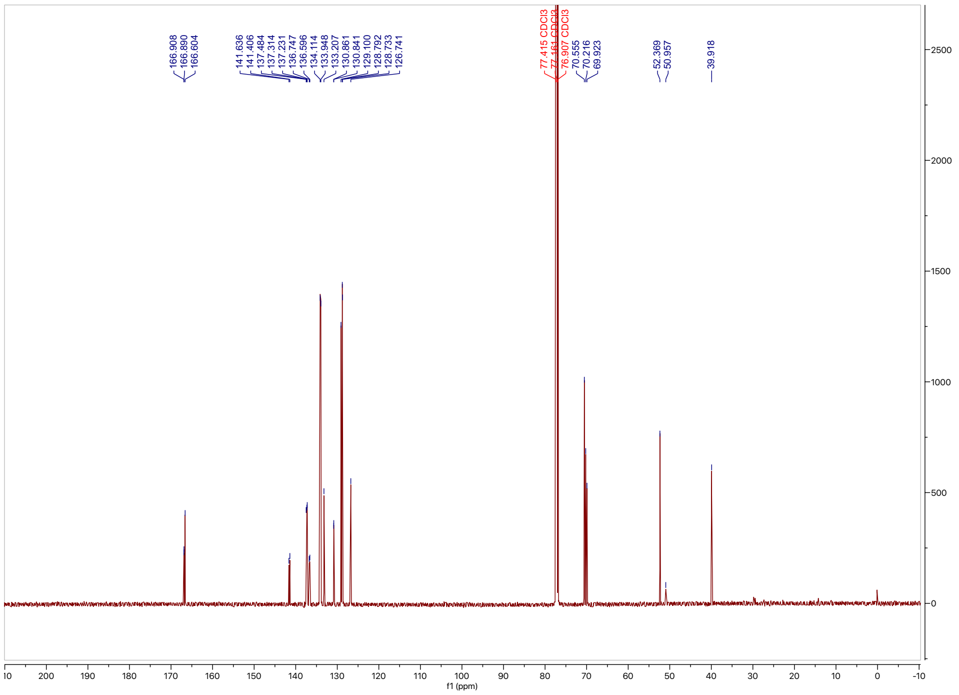

Figure S31. NMR spectra of 6b. **A)** Structure of **6b**. **B)** ^{1}H-NMR (500 MHz, $CDCl_3$) spectrum of **6b**. **C)** ^{13}C NMR (126 MHz, $CDCl_3$) spectrum of **6b**.

A)

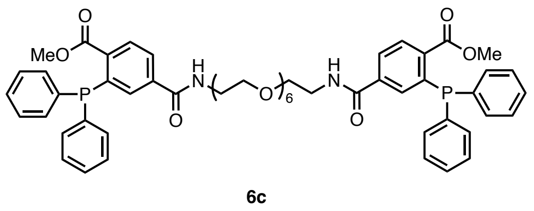

B)

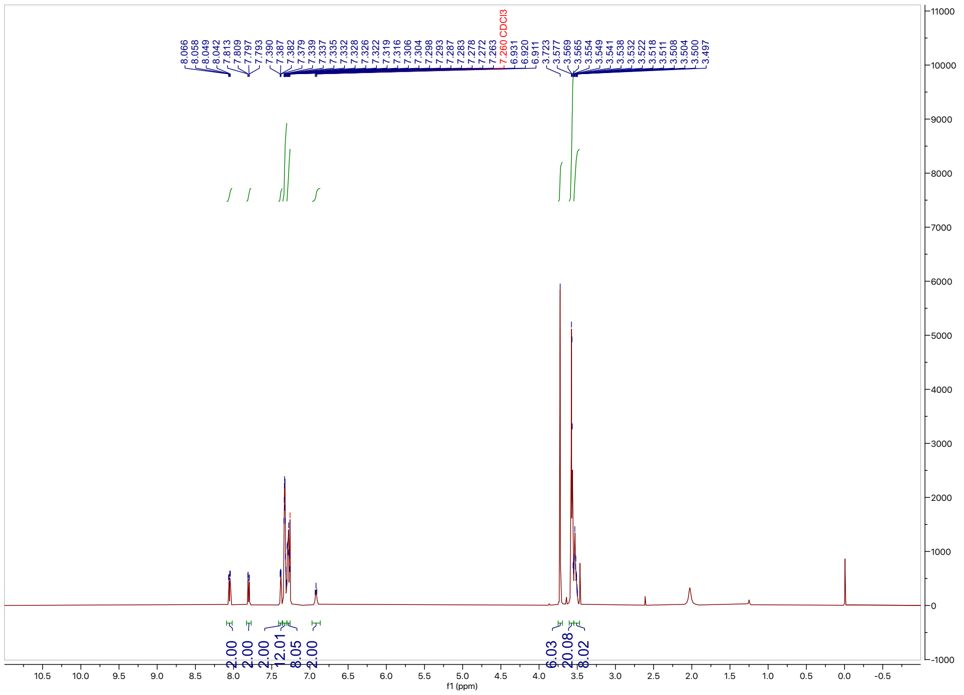

C)

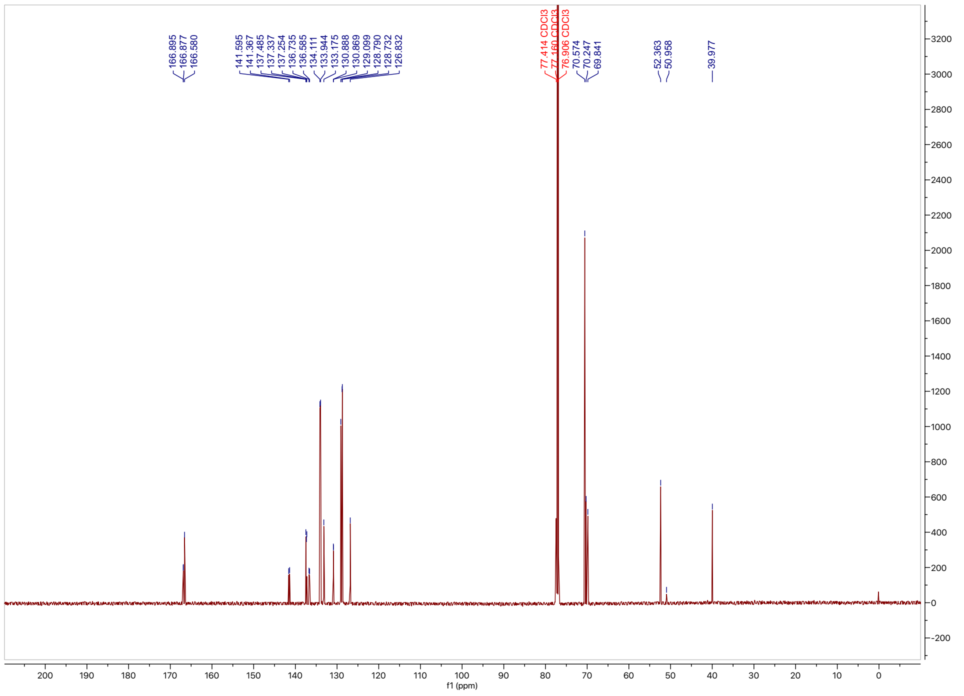

Figure S32. NMR spectra of 6c. **A)** Structure of **6c**. **B)** ^{1}H-NMR (500 MHz, $CDCl_3$) spectrum of **6c**. **C)** ^{13}C NMR (126 MHz, $CDCl_3$) spectrum of **6c**.

A)

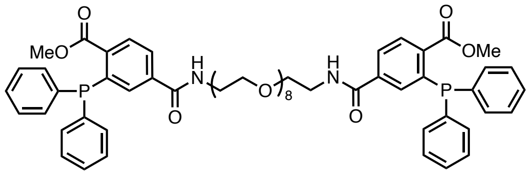

6d

B)

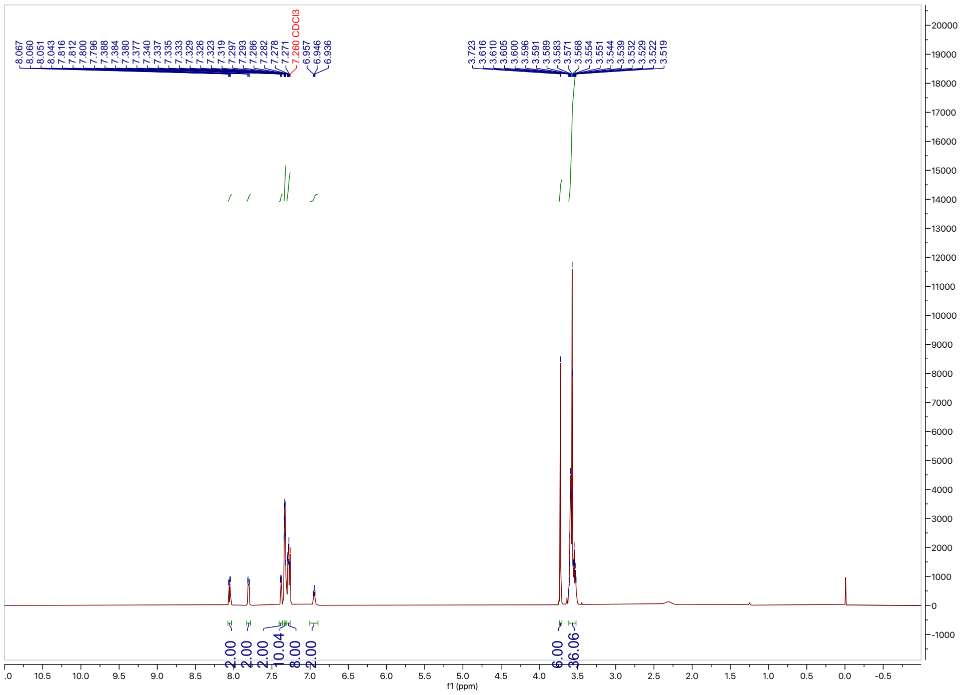

C)

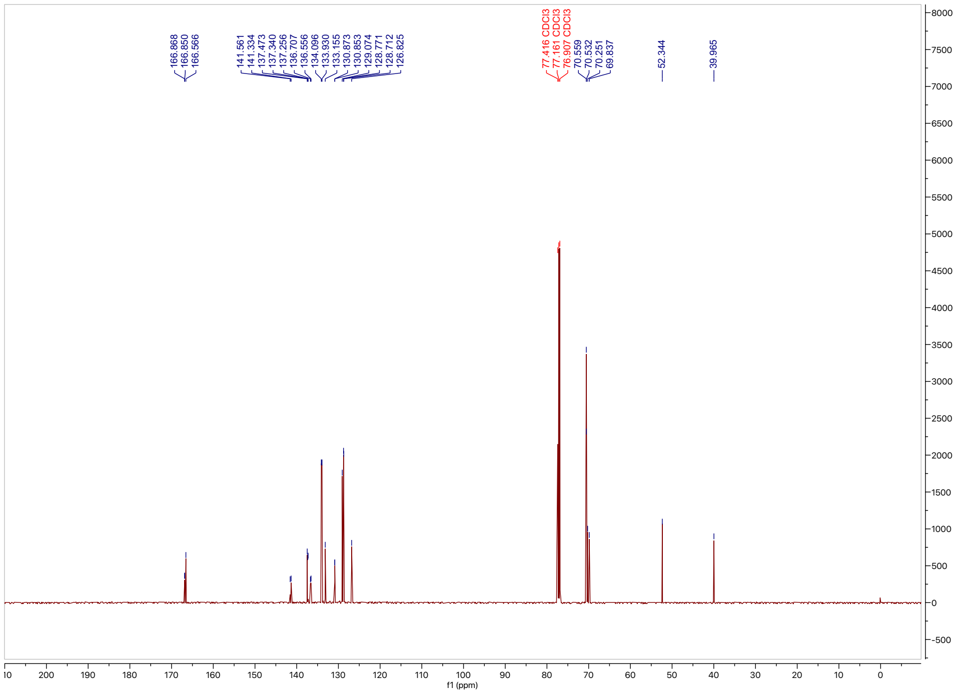

Figure S33. NMR spectra of 6d. **A)** Structure of **6d**. **B)** ^{1}H-NMR (500 MHz, $CDCl_3$) spectrum of **6d**. **C)** ^{13}C NMR (126 MHz, $CDCl_3$) spectrum of **6d**.

References

1. Saxon, E.; Bertozzi, C. R. Cell surface engineering by a modified Staudinger reaction. *Science* **2000**, *287*, 2007–10.

2. Sun, Y.; Lau, S. Y.; Lim, Z. W.; Chang, S. C.; Ghadessy, F.; Partridge, A.; Miserez, A. Phase-separating peptides for direct cytosolic delivery and redox-activated release of macromolecular therapeutics. *Nat. Chem.* **2022**, *14*, 274–283.

3. Wang, J.; Wolf, R. M.; Caldwell, J. W.; Kollman, P. A.; Case, D. A. Development and testing of a general amber force field. *J. Comput. Chem.* **2004***, 25,* 1157–1174.

4. Sousa da Silva, A.W.; Vranken, W. F. ACPYPE - AnteChamber PYthon Parser interfacE. *BMC Res Notes* **2012**, *5*, 367.

5. Vanquelef, E.; Simon, S.; Marquant, G.; Garcia, E.; Klimerak, G.; Delepine, J. C.; Cieplak, P.; Dupradeau, F.-Y.. R.E.D. Server: a web service for deriving RESP and ESP charges and building force field libraries for new molecules and molecular fragments. *Nucleic Acids Res.* **2011**, *39*, W511–W517.

6. Bayly, C. I.; Cieplak, P.; Cornell, W.; Kollman, P. A. A well-behaved electrostatic potential based method using charge restraints for deriving atomic charges: the RESP model. *J. Phys. Chem*. **1993**, *97*, 10269–10280.

7. Jorgensen, W. L.; Chandrasekhar, J.; Madura, J. D.; Impey, R. W.; Klein, M. L. Comparison of simple potential functions for simulating liquid water. *J. Chem. Phys.* **1983**, *79*, 926–935.

8. Abraham, M. et al. Gromacs 2023.1 manual. https://doi.org/10.5281/zenodo.7852189.

9. Bennett, C. H. Efficient estimation of free energy differences from Monte Carlo data. *J. Comput. Phys*. **1976**, *22*, 245-268.

10. Bussi, G.; Donadio, D.; Parrinello, M. Canonical sampling through velocity rescaling. *J. Chem. Phys.* **2007**, *126*, 014101.

11. Parrinello, M.; Rahman, A. Polymorphic transitions in single crystals: a new molecular dynamics method. *J. Appl. Phys.* **1981**, *52*, 7182–7190.
